# Sparse identification of contrast gain control in the fruit fly photoreceptor and amacrine cell layer

**DOI:** 10.1186/s13408-020-0080-5

**Published:** 2020-02-12

**Authors:** Aurel A. Lazar, Nikul H. Ukani, Yiyin Zhou

**Affiliations:** Department of Electrical Engineering, Columbia University, New York, USA

**Keywords:** Sparse functional identification, Contrast gain control, Fruit fly, Photoreceptor, Divisive normalization

## Abstract

The fruit fly’s natural visual environment is often characterized by light intensities ranging across several orders of magnitude and by rapidly varying contrast across space and time. Fruit fly photoreceptors robustly transduce and, in conjunction with amacrine cells, process visual scenes and provide the resulting signal to downstream targets. Here, we model the first step of visual processing in the photoreceptor-amacrine cell layer. We propose a novel divisive normalization processor (DNP) for modeling the computation taking place in the photoreceptor-amacrine cell layer. The DNP explicitly models the photoreceptor feedforward and temporal feedback processing paths and the spatio-temporal feedback path of the amacrine cells. We then formally characterize the contrast gain control of the DNP and provide sparse identification algorithms that can efficiently identify each the feedforward and feedback DNP components. The algorithms presented here are the first demonstration of tractable and robust identification of the components of a divisive normalization processor. The sparse identification algorithms can be readily employed in experimental settings, and their effectiveness is demonstrated with several examples.

## Introduction

Sensory processing systems in the brain extract relevant information from stimuli whose amplitude can vary orders of magnitude [1–4]. Consequently, at each layer of processing, starting right from sensory transduction, neurons need to map their output into a range that can be effectively processed by subsequent neural circuits. As an example, photoreceptors [5–8] and olfactory receptor neurons [9, 10] in both vertebrates and invertebrates respectively adapt to a large range of intensity/temporal contrast values of visual and odorant stimuli. Adaptation to mean and variance of the stimuli has been observed in the auditory system [11, 12] as well. Further down the visual pathway, motion sensitive neurons in vertebrates and invertebrates have been shown to be robust at various brightness and contrast levels [13–15].

Early visual circuits, such as the photoreceptor/amacrine cell layer of the fruit fly brain, are believed to perform spatio-temporal intensity and contrast gain control for dynamic adaptation to visual stimuli whose intensity and contrast levels vary orders of magnitude both in space and time. The mechanism underlying temporal and spatio-temporal contrast gain control in the vertebrate retina [16–20] has often been characterized as a change of the receptive fields in response to changing stimulus statistics [21]. However, while linear receptive fields can be estimated at each transition between different gain values, characterizing the full dynamics of contrast gain control received little attention. Current theoretical methods for describing spatio-temporal gain control lack a systematic framework for characterizing its dynamics, and identification algorithms to estimate circuit components are generally not available.

One exception is the use of theory of dynamical systems for modeling nonlinear gain control. Examples include the Volterra series expansion [22, 23] and the nonlinear autoregressive moving average model with exogenous inputs (NARMAX) [24, 25]. Both formalisms exhibit extensive rigorous tools of functional identification. However, the former typically requires high-order Volterra kernels to model the highly nonlinear gain control, and its identification suffers from the curse of dimensionality. In practice, using the second- or third-order Volterra kernels is computationally not tractable with commodity hardware, while at the same time not fully capturing the dynamics of gain control. For the latter, an extension to the spatio-temporal domain is often out of reach.

Divisive normalization provides an alternative nonlinear operator to model gain control. Divisive normalization [26] has been proposed as a canonical circuit model of computation for many sensory processing circuits underlying adaptation and attention [4, 27–31]. Divisive normalization models in sensory systems are often associated with a population of neurons where each receives inputs from the pool [26, 32]. Recent studies have shown that divisive normalization is a suitable candidate for describing the contrast gain control and its dynamics [33].

Existing modeling studies of divisive normalization in sensory systems mostly focus on establishing a connection between gain control and the statistical properties of natural sensory stimuli [28, 34–36]. There is a lack of general mathematical framework for identifying divisive normalization circuits from recorded data.

Early models of divisive normalization [27] and their derivatives [26, 28, 29, 37–40] only consider feedforward divisive normalization circuits. The modeled neural circuits often exhibit, however, extensive feedback circuits [22, 41].

In this paper, we address the above issues by modeling the photoreceptor/amacrine cells layer of the fruit fly as a multi-input multi-output (MIMO) feedforward and feedback temporal and spatio-temporal divisive normalization processor (DNP). The MIMO DNPs are built upon temporal and spatio-temporal feedforward and feedback of divisive normalization operators constructed from low-dimensional Volterra operators. Combining with sparse identification methods [42], we provide efficient algorithms for identifying all the components of the temporal as well as spatio-temporal divisive normalization processors.

This manuscript is organized as follows. In Sect. 2 the overall architecture of the divisive normalization processor (DNP) is introduced and its power of modeling contrast gain control is demonstrated. We first describe in Sect. 2.1 the biological model of photoreceptor-amacrine cell layer. In Sect. 2.2, we introduce a general model for divisive normalization in the time domain. The temporal DNP consists of the ratio of two nonlinear functionals acting on the input stimulus. In Sect. 2.3 we then extend the model to space-time domain to include models of lateral feedback from amacrine cells and demonstrate its processing power. In Sects. 3 and 4, we provide identification algorithms and show that the temporal and spatio-temporal DNPs can be efficiently identified. We demonstrate the effectiveness of the algorithms with several examples. We conclude the paper with a discussion in Sect. 5.

## The architecture of divisive normalization processors

In Sect. 2.1 we start by motivating the present work. We then introduce the architecture of divisive normalization processors in the time domain (Sect. 2.2) and space-time domain (Sect. 2.3). Finally, in Appendix 1 we provide examples that characterize the I/O mapping of the class of temporal and spatio-temporal divisive normalization processors previously described.

### Modeling the photoreceptors and amacrine cells layer

In what follows, we anchor the model description around the photoreceptor-amacrine cell layer of the fruit fly. The fly retina consists of ∼800 ommatidia, each of which hosts photoreceptors whose axons terminate in a secondary neuropil called lamina. There, they provide inputs to columnar large monopolar cells (LMCs) that project to the third visual neuropil, and to amacrine cells [43]. Amacrine cells are interneurons that innervate axon terminals of multiple photoreceptors. The photoreceptors, in turn, receive lateral feedback from the amacrine cells as well as feedback from LMCs such as L2 neurons [41, 44, 45].

**Figure 1:**
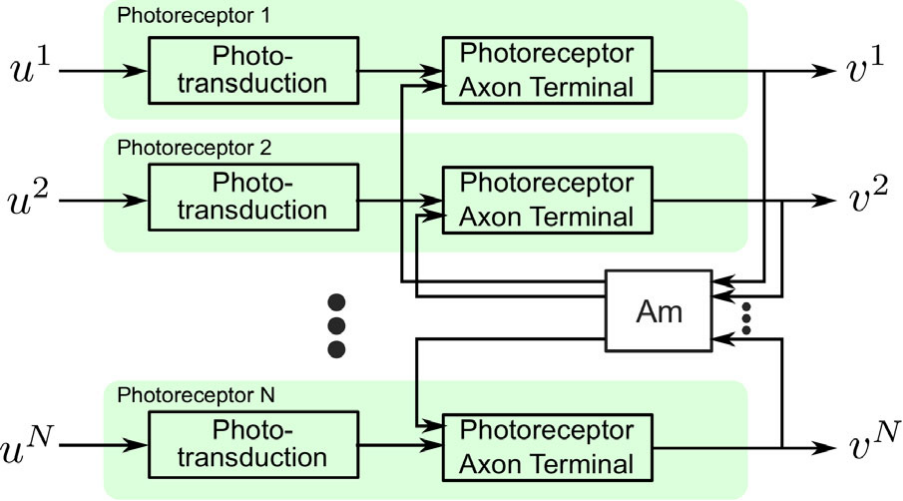
A schematic diagram of interaction between Amacrine cells and photoreceptors in multiple cartridges

A circuit diagram of the photoreceptor-amacrine cell layer is shown in Fig. 1. For the sake of clarity, we assume here that an ommatidium consists of a single photoreceptor. It has been shown that the outputs of photoreceptors exhibit rapid gain control through both the phototransduction process and the interaction with such feedback loops [25, 46–49].

In what follows we propose a model comprising nonlinear transformations combined with divisive normalization that can model such gain control and can account for diverse dynamics. We will also show that the model we propose can be systematically identified from observed input output pairs.

### Divisive normalization processors in the time domain

In this section we present the modeling of temporal stimuli in 2.2.1 and introduce a class of temporal divisive normalization processors for modeling photoreceptors in Sect. 2.2.2.

#### Modeling temporal stimuli

We model the temporal varying stimuli $u_{1}=u_{1}(t)$, $t \in \mathbb{D} \subseteq {\mathbb {R}}$, to be real-valued elements of the space of trigonometric polynomials [50]. The choice of the space of the trigonometric polynomials has, as we will see, substantial computational advantages. A temporal stimulus models the visual field arising at the input of a single photoreceptor.

##### Definition 1

The space of trigonometric polynomials ${\mathcal {H}}_{1}$ is the Hilbert space of complex-valued functions

1 $$ u(t) = \sum_{l=-L}^{L} a_{l} \cdot e_{l}(t), $$

over the domain $\mathbb{D} = [0, S]$, where

2 $$ e_{l}(t) = \frac{1}{\sqrt{S}}\operatorname{exp} \biggl( \frac{jl\varOmega }{L}t \biggr). $$

Here, *Ω* denotes the bandwidth and *L* the order of the space. Stimuli $u_{1}\in {\mathcal {H}}_{1}$ are extended to be periodic over ${\mathbb {R}}$ with period $S = \frac{2\pi L}{\varOmega }$.

${\mathcal {H}}_{1}$ is a reproducing kernel Hilbert space (RKHS) [51] with reproducing kernel (RK)

3 $$ K_{1}\bigl(t;t'\bigr) = \sum _{l=-L}^{L}e_{l}\bigl(t-t' \bigr). $$

RKHSs have been previously employed for modeling neural encoding of temporal, auditory, and visual stimuli [52–55].

We denote the dimension of ${\mathcal {H}}_{1}$ by $\dim ({\mathcal {H}}_{1})$ and $\dim ({\mathcal {H}}_{1}) = 2L+1$.

##### Definition 2

The tensor product space ${\mathcal {H}}_{2}={\mathcal {H}}_{1} \otimes {\mathcal {H}}_{1}$ is an RKHS with reproducing kernel

4 $$ K_{2}\bigl(t_{1},t_{2};t'_{1},t'_{2} \bigr) = \sum_{l_{1}=-L}^{L}\sum _{l_{2}=-L}^{L}e_{l_{1}}\bigl(t_{1}-t'_{1} \bigr) \cdot e_{l_{2}}\bigl(t_{2}-t'_{2} \bigr). $$

Note that $\dim ({\mathcal {H}}_{2}) = \dim ({\mathcal {H}}_{1})^{2} = (2 L +1)^{2}$.

#### Temporal divisive normalization processors

**Figure 2:**
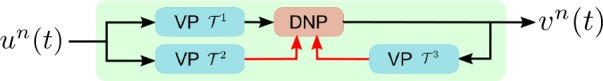
Schematic diagram of a temporal divisive normalization processor

We first consider single photoreceptors without feedback from the amacrine cells shown in Fig. 1. A schematic of the temporal divisive normalization processor (DNP) modeling the photoreceptor is shown in Fig. 2. For notational simplicity, we consider a single photoreceptor here. The input visual stimulus to the photoreceptor is denoted by $u=u(t), t\in \mathbb{D}$, and the output electric current by $v=v(t), t\in \mathbb{D}$.

##### Remark 1

Note that, in a single photoreceptor, photons are first absorbed by a large number of microvilli [47] (not shown). Microvilli generate “quantum bumps” in response to photons; the photoreceptor aggregates the bumps and in the process creates the transduction current. Calcium ion influx and calcium diffusion into the photoreceptor cell body may change the sensitivity of the transduction cascade. A high concentration of calcium (buffer) can result in a photon to be ineffective and may also affect the duration and the magnitude of quantum bumps [56].

The DNP consists of (1) a feedforward Volterra processor (VP) $\mathcal{T}^{1}$, (2) a feedforward normalization VP $\mathcal{T}^{2}$, and (3) a feedback normalization VP $\mathcal{T}^{3}$. The output of the photoreceptor amounts to

5 $$ v^{n} = \frac{\mathcal{T}^{1} u^{n}}{\mathcal{T}^{2} u^{n} + \mathcal{T}^{3} v^{n}}, \quad n=1,2,\ldots ,N, $$

where

6 $$\begin{aligned} \bigl(\mathcal{T}^{l} u^{n}\bigr) (t) =& b^{l} + \int _{\mathbb{D}} h_{1}^{l}(s) u^{n}(t-s) \,ds \\ &{}+ \int _{\mathbb{D}^{2}} h_{2}^{l}(s_{1},s_{2})u^{n}(t-s_{1})u^{n}(t-s_{2})\,ds_{1}\,ds_{2},\quad l = 1,2, \end{aligned}$$

and

7 $$ \bigl(\mathcal{T}^{3} v^{n}\bigr) (t) = b^{3} + \int _{\mathbb{D}} h_{1}^{3} (s) v^{n}(t-s) \,ds + \int _{\mathbb{D}^{2}} h_{2}^{3} (s_{1},s_{2}) v^{n}(t-s_{1})v^{n}(t-s_{2})\,ds_{1}\,ds_{2} . $$

Here, $b^{l}$, $l=1,2,3$, are the zeroth-order Volterra kernels (constants), $h_{1}^{l}(t)$, $l=1,2,3$, are first-order Volterra kernels (impulse responses of linear filters), and $h_{2}^{l}(t,s)$, $l=1,2,3$, are second-order Volterra kernels. As before, $\mathbb{D}$ denotes the domain of the input space, and $\mathbb{D}^{2}=\mathbb{D}\times \mathbb{D}$.

##### Remark 2

For the sake of tractability, we limit each nonlinear functional in (6) and (7) to be composed of only first- and second-order Volterra kernels. The division in (5) allows us, however, to model nonlinear processing of much higher orders.

We note that *v* in (5) is invariant under scaling by the same factor of the numerator and denominator. Hence, without loss of generality, we will assume $b^{2}+b^{3}=1$. We will also assume that the DNP is bounded-input bounded-output [57].

#### Modeling temporal DNP feedback filters

Here, we define the filter kernels in equations (6) and (7).

##### Definition 3

Let $h^{l}_{p} \in \mathbb{L}^{1}(\mathbb{D}^{p})$, $l=1,2$, $p=1,2$, where $\mathbb{L}^{1}$ denotes the space of Lebesgue integrable functions. The operator $\mathcal{P}_{1}: \mathbb{L}_{1}(\mathbb{D}) \rightarrow {\mathcal {H}}_{1}$ given by

8 $$ \bigl(\mathcal{P}_{1} h^{l}_{1}\bigr) (t) = \int _{\mathbb{D}} h^{l}_{1} \bigl(t'\bigr) K_{1}\bigl(t; t'\bigr) \,dt' $$

is called the projection operator from $\mathbb{L}^{1}(\mathbb{D})$ to ${\mathcal {H}}_{1}$. Similarly, the operator $\mathcal{P}_{2}: \mathbb{L}_{1}(\mathbb{D}^{2}) \rightarrow {\mathcal {H}}_{2}$ given by

9 $$ \bigl(\mathcal{P}_{2}h^{l}_{2}\bigr) (t_{1};t_{2}) = \int _{\mathbb{D}^{2}} h^{l}_{2} \bigl(t'_{1}; t'_{2}\bigr) K_{2}\bigl(t_{1},t_{2}; t'_{1},t'_{2} \bigr) \,dt'_{1}\,dt'_{2} $$

is called the projection operator from $\mathbb{L}^{1}(\mathbb{D}^{2})$ to ${\mathcal {H}}_{2}$.

Note that, for $u^{n} \in {\mathcal {H}}_{1}, \mathcal{P}_{1} u^{n} = u^{n}$. Moreover, with $u_{2}^{n}(t_{1},t_{2}) = u^{n}(t_{1})u^{n}(t_{2})$, $\mathcal{P}_{2} u_{2}^{n} = u_{2}^{n}$. Thus,

10 $$ \begin{aligned}[b] \bigl(\mathcal{T}^{l} u^{n} \bigr) (t) ={} &b^{l} + \int _{\mathbb{D}} \bigl(\mathcal{P}_{1} h_{1}^{l}\bigr) (s) u^{n}(t-s) \,ds \\ &{} + \int _{\mathbb{D}^{2}} \bigl(\mathcal{P}_{2} h_{2}^{l}\bigr) (s_{1},s_{2})u^{n}(t-s_{1})u^{n}(t-s_{2})\,ds_{1}\,ds_{2}, \quad l = 1,2, \end{aligned} $$

and by assuming that $h^{l}_{1} \in {\mathcal {H}}_{1}$, $l=1,2$, and $h^{l}_{2} \in {\mathcal {H}}_{2}$ we recover the simple form of equation (6).

We model the output waveforms $v^{o} = v^{o} (t)$, $t \in \mathbb{D} \subseteq {\mathbb {R}}$, to be real-valued elements of the space of trigonometric polynomials [50].

##### Definition 4

The space of trigonometric polynomials ${\mathcal {H}}_{1}^{o}$ is the Hilbert space of complex-valued functions

11 $$ v^{o}(t) = \sum_{l=-L^{o}}^{L^{o}} a_{l}^{o} \cdot e_{l}^{o} (t), $$

over the domain $\mathbb{D} = [0, S]$, where

12 $$ e_{l}^{o} (t) = \frac{1}{\sqrt{S^{o}}}\operatorname{exp} \biggl(\frac{jl\varOmega ^{o}}{L^{o}}t \biggr). $$

Here, $\varOmega ^{o}$ denotes the bandwidth and $L^{o}$ the order of the space. The output waveforms $v^{o}\in {\mathcal {H}}_{1}^{o}$ are extended to be periodic over ${\mathbb {R}}$ with period $S^{o} = \frac{2\pi L^{o}}{\varOmega ^{o}}$.

${\mathcal {H}}_{1}^{o}$ is a reproducing kernel Hilbert space (RKHS) [51] with reproducing kernel (RK)

13 $$ K_{1}^{o}\bigl(t;t'\bigr) = \sum _{l=-L^{o}}^{L^{o}} e_{l}^{o} \bigl(t-t'\bigr). $$

We denote the dimension of ${\mathcal {H}}_{1}^{o}$ by $\dim ({\mathcal {H}}_{1}^{o})$ and $\dim ({\mathcal {H}}_{1}^{o}) = 2L^{o}+1$.

##### Definition 5

The tensor product space ${\mathcal {H}}_{2}^{o} = {\mathcal {H}}_{1}^{o} \otimes {\mathcal {H}}_{1}^{o}$ is an RKHS with reproducing kernel

14 $$ K_{2}^{o}\bigl(t_{1},t_{2};t'_{1},t'_{2} \bigr) = \sum_{l_{1}=-L^{o}}^{L^{o}}\sum _{l_{2}=-L^{o}}^{L^{o}}e_{l_{1}}^{o} \bigl(t_{1}-t'_{1}\bigr) \cdot e_{l_{2}}^{o}\bigl(t_{2}-t'_{2} \bigr). $$

Note that $\dim ({\mathcal {H}}_{2}^{o}) = \dim ({\mathcal {H}}_{1}^{o})^{2} = (2 L^{o} +1)^{2}$.

##### Definition 6

Let $h^{3}_{p} \in \mathbb{L}^{1}(\mathbb{D}^{p}), p=1,2$, where $\mathbb{L}^{1}$ denotes the space of Lebesgue integrable functions. The operator $\mathcal{P}_{1}^{o}: \mathbb{L}_{1}(\mathbb{D}) \rightarrow {\mathcal {H}}_{1}^{o}$ given by

15 $$ \bigl(\mathcal{P}_{1}^{o} h^{3}_{1} \bigr) (t) = \int _{\mathbb{D}} h^{3}_{1} \bigl(t'\bigr) K_{1}^{o}\bigl(t; t'\bigr) \,dt' $$

is called the projection operator from $\mathbb{L}^{1}(\mathbb{D})$ to ${\mathcal {H}}_{1}$. Similarly, the operator $\mathcal{P}_{2}^{o}: \mathbb{L}_{1}(\mathbb{D}^{2}) \rightarrow {\mathcal {H}}^{o}_{2}$ given by

16 $$ \bigl(\mathcal{P}_{2}^{o} h^{3}_{2} \bigr) (t_{1};t_{2}) = \int _{\mathbb{D}^{2}} h^{3}_{2} \bigl(t'_{1}; t'_{2}\bigr) K_{2}^{o}\bigl(t_{1},t_{2}; t'_{1},t'_{2}\bigr) \,dt'_{1}\,dt'_{2} $$

is called the projection operator from $\mathbb{L}^{1}(\mathbb{D}^{2})$ to ${\mathcal {H}}_{2}^{o}$.

We note that

17 $$\begin{aligned} \bigl(\mathcal{T}^{3} v^{n}\bigr) (t) =& b^{3} + \int _{\mathbb{D}} \bigl(\mathcal{P}_{1}^{o} h_{1}^{3}\bigr) (s) v^{n}(t-s) \,ds \\ &{}+ \int _{\mathbb{D}^{2}} \bigl(\mathcal{P}_{2}^{o} h_{2}^{3}\bigr) (s_{1},s_{2}) v^{n}(t-s_{1})v^{n}(t-s_{2})\,ds_{1}\,ds_{2} . \end{aligned}$$

If we now assume that $h^{3}_{1}\in {\mathcal {H}}^{o}_{1}$ and $h^{3}_{2} \in {\mathcal {H}}^{o}_{2}$, then $\mathcal{P}_{1}^{o} h_{1}^{3} = h_{1}^{3}$ and $\mathcal{P}_{2}^{o} h_{2}^{3} = h_{2}^{3}$, respectively, and the above equation is identical with equation (7) above.

### Divisive normalization processors in the space-time domain

**Figure 3:**
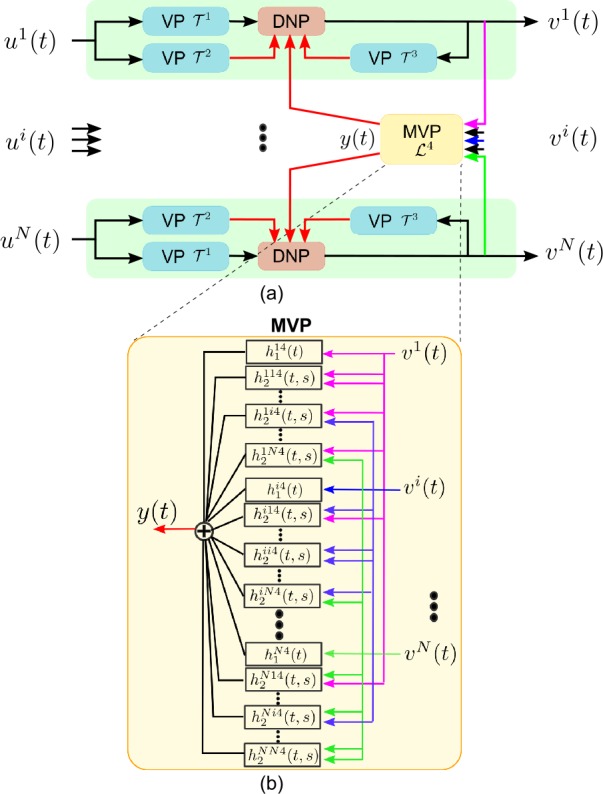
Schematic block diagram of the spatio-temporal divisive normalization processor

In Sect. 2.2, we described a temporal divisive normalization processor model. The normalization term was the sum of a processed version of the input and the output. However, many biological circuits are thought to exhibit lateral inhibition and gain control [26, 58–61]. An example is provided by the photoreceptor-amacrine cell layer shown in Fig. 1. In Fig. 3, we provide a model of the schematic in Fig. 1. This model is a ‘circuit’ extension of the one shown in Fig. 2 and accounts explicitly for lateral inhibition. We anchor the extension in the visual system where the input domain is spatio-temporal.

In Sect. 2.3.1 we model spatio-temporal stimuli and in Sect. 2.3.2 the spatio-temporal divisive normalization processors.

#### Modeling spatio-temporal stimuli

In this section we provide a model of the interaction between a group of photoreceptors and an amacrine cell. In each photoreceptor, the phototransduction process converts light into current and excites the membrane of the photoreceptor. The voltage signal is then propagated through its axon to the lamina. While photoreceptors provide inputs to amacrine cells, their axon terminals also receive amacrine cell input. Since an amacrine cell innervates multiple lamina cartridges, it provides spatial feedback to several photoreceptors in a small neighborhood.

We extend the temporal divisive normalization processor depicted in Fig. 2 to process spatio-temporal stimuli as shown in Fig. 3. Each spatially sampled point, or pixel, denoted as $u^{i}(t), i=1,2,\ldots ,N$, is first processed by a temporal DNP. For simplicity rather than picking different Volterra kernels for each branch, $\mathcal{T}^{1}, \mathcal{T}^{2}$, and $\mathcal{T}^{3}$ are shared across branches. In addition, we introduce a multi-input Volterra processor (MVP) to model the spatio-temporal feedback due to the amacrine cell. Each of the branches in Fig. 3, without the input of the MVP block, is equivalent to the model in Fig. 2.

#### Spatio-temporal divisive normalization processors

As shown in Fig. 3(b), MVPs are comprised of second-order filters acting on DNP output pairs in addition to linear filters independently acting on each DNP output. Thus, the inputs to the MVP are the DNP outputs $v^{i}(t), i = 1,2,\ldots ,N$, and the MVP output amounts to

18 $$ \begin{aligned}[b] \bigl(\mathcal{L}^{4}\mathbf{v} \bigr) (t) = {}& b^{4} + \sum_{i=1}^{N} \biggl( \int _{\mathbb{D}} h^{i4}_{1} (s)v^{i}(t-s)\,ds \biggr) \\ &{} + \sum_{i=1}^{N}\sum _{j=1}^{N} \biggl( \int _{\mathbb{D}^{2}} h_{2}^{ij4} (s_{1},s_{2})v^{i}(t-s_{1})v^{j}(t-s_{2})\,ds_{1}\,ds_{2} \biggr) , \end{aligned} $$

where

19 $$ \mathbf{v}(t) = \bigl[v^{1}(t), v^{2}(t), \ldots , v^{N}(t) \bigr]^{T}, $$

$b^{4}$ is the zeros-th-order Volterra kernel (constant). Furthermore, $h^{i4}_{1}\in {\mathcal {H}}^{o}_{1}$, $i=1,2,\ldots ,N$, are the first-order Volterra kernels whose inputs are $v^{i}$, $i=1,2,\ldots ,N$, respectively, and $h^{ij4}_{2}\in {\mathcal {H}}^{o}_{2}$, $i,j=1,2,\ldots ,N$, are the second-order Volterra kernels whose inputs are the pairs $(v^{i}, v^{j})$, $i,j=1,2,\ldots ,N$, respectively.

The full model in Fig. 3 thus consists of parallel channels as depicted in Fig. 2 with the added cross-channel feedback normalization/gain control provided by the MVP block.

The overall output of the spatio-temporal DNP can be expressed as

20 $$ v^{n}(t) = \frac{\mathcal{T}^{1} u^{n}}{\mathcal{T}^{2} u^{n} + \mathcal{T}^{3} v^{n} + \mathcal{L}^{4} \mathbf{v}},\quad n=1,2,\ldots ,N. $$

W.L.O.G., we assume that $b^{2} + b^{3} + b^{4} = 1$.

#### Spatio-temporal DNPs and contrast gain control

The relationship and some intuition behind the modeling power of spatio-temporal DNPs are provided in several examples in Appendix 1. The I/O of three simple spatio-temporal DNPs stimulated with different inputs is briefly mentioned here. In the first example, we evaluated the response of a DNP with four photoreceptors under different background light intensity levels. In Fig. 9 one of the photoreceptors is subjected to an additional flash of different light intensity, while the inputs of the other three are kept at the same background level. The steady state response of the photoreceptor that receives the additional flash is shifted as a function of the background-intensity level. In the second example, contrast gain control exerted by the amacrine cells is demonstrated for the same DNP in Fig. 10. The effect of the MVP block on the RMS contrast can be clearly seen in Fig. 10 and is quantitatively evaluated in Fig. 11. Finally, an example of steady state I/O visualization of a natural image with resolution of $1536\times 1024$ pixels at low, medium, and high luminance values is shown in Fig. 13. The image was divided into $16\times 16$ spatio-temporal DNP blocks with a four-pixel overlap in each direction.

## Sparse identification of temporal DNPs

In what follows we derive sparse identification algorithms for the components of spatio-temporal DNPs depicted in Fig. 2 and formally defined in equation (5). In what follows, we assume that during experimental trials, the single isolated photoreceptor *n* is presented with *M* test stimuli $u^{nm} = u^{nm}(t)$, $m=1, 2, \ldots ,M$, and for each trial the outputs $v^{nm} = v^{nm}(t)$, $m=1, 2, \ldots , M$, are recorded. The objective is to identify the model components $b_{1}, h^{l}_{1}, h^{l}_{2}$, $l = 1,2,3$, from the knowledge of the inputs and outputs.

### Deriving the sparse identification algorithm for temporal DNPs

#### Lemma 1

*WithMinput stimuli presented to a temporal DNP*, *let the inputs*$u^{nm}(t)$*and the outputs*$v^{nm}(t)$*be sampled at times*$(t_{k})$, $k = 1,2,\ldots ,T$. *Then*

21 $$ \begin{aligned}[b] &b^{1} + \bigl\langle h_{1}^{1}, \phi _{11}^{nmk} \bigr\rangle _{{\mathcal {H}}_{1}} + \bigl\langle h_{1}^{2} , \phi _{12}^{nmk} \bigr\rangle _{{\mathcal {H}}_{1}} + \bigl\langle h_{1}^{3} , \phi _{13}^{nmk} \bigr\rangle _{{\mathcal {H}}_{1}^{o}} \\ &\quad {}+ \bigl\langle h_{2}^{1}, \phi _{21}^{nmk} \bigr\rangle _{{\mathcal {H}}_{2}} + \bigl\langle h_{2}^{2}, \phi _{22}^{nmk} \bigr\rangle _{{\mathcal {H}}_{2}} + \bigl\langle h_{2}^{3}, \phi _{23}^{nmk} \bigr\rangle _{{\mathcal {H}}_{2}^{o}} = q^{nmk} , \end{aligned} $$

*where the sampling functions*$\phi _{11}^{nmk} \in {\mathcal {H}}_{1}$, $\phi _{12}^{nmk}\in \mathcal{H}_{1}$, $\phi _{13}^{nmk}\in \mathcal{H}_{1}^{o}$, $\phi _{21}^{nmk}\in \mathcal{H}_{2}$, $\phi _{22}^{nmk}\in \mathcal{H}_{2}$, *and*$\phi _{23}^{nmk}\in \mathcal{H}_{2}^{o}$*are given by*

22 $$\begin{aligned} \begin{aligned} &\phi _{11}^{nmk} (t)= u^{nm}(t_{k}-t) , \\ &\phi _{12}^{nmk} (t)= -q^{nm}(t_{k}) u^{nm}(t_{k}-t) , \\ &\phi _{13}^{nmk} (t)= -q^{nm}(t_{k}) \bigl(\mathcal{P}_{1}^{o} v^{nm}\bigr) (t_{k}-t) , \\ &\phi _{21}^{nmk} (t, s)= u^{nm}(t_{k}-t)u^{nm}(t_{k}-s) , \\ &\phi _{22}^{nmk} (t, s)= -q^{nm}(t_{k}) u^{nm}(t_{k}-t)u^{nm}(t_{k}-s) , \\ &\phi _{23}^{nmk} (t, s)= -q^{nm}(t_{k}) \bigl(\mathcal{P}_{1}^{o} v^{nm}\bigr) (t_{k}-t) \bigl(\mathcal{P}_{1}^{o} v^{nm}_{1}\bigr) (t_{k}-s) , \end{aligned} \end{aligned}$$

*and*$q^{nmk} = v^{nm}(t_{k})$*for all*$m=1,2,\ldots,M$*and*$k=1,2,\ldots,T$.

#### Proof

See Appendix 2. □

#### Remark 3

From (21), it can be seen that the identification of the divisive normalization processor has been reformulated as a generalized sampling problem [62] in ${\mathbb {R}}\oplus {\mathcal {H}}_{1}\oplus {\mathcal {H}}_{1}\oplus {\mathcal {H}}_{1}^{o}\oplus {\mathcal {H}}_{2}\oplus {\mathcal {H}}_{2}\oplus {\mathcal {H}}_{2}^{o}$. Subsequently, the divisive normalization model can be identified by solving a system of linear equations.

In order to solve the system of linear equations in (21), we rewrite them first in matrix form.

#### Lemma 2

*Equation* (21) *can be expressed in matrix form as follows*:

23 $$ \mathbf{c}_{1}^{\mathsf {T}}\boldsymbol{\varPhi}^{nmk} + \operatorname{Tr} \bigl(\mathbf{C}_{2}^{\mathsf {H}}\boldsymbol {\varXi}^{nmk} \bigr) = q^{nmk} $$

*for all*$m=1,2,\ldots ,M$*and*$k=1,2,\ldots ,T$, *where*$\operatorname{Tr}(\cdot )$*denotes the trace operator*, *and*

24 $$\begin{aligned} &\textit{Measurements} \leftarrow q^{nmk}, (scalar) \end{aligned}$$

25 $$\begin{aligned} &\textit{Unknowns} \leftarrow \textstyle\begin{cases} \mathbf{c}_{1} = \begin{bmatrix} b^{1} & (\mathbf{h}^{1}_{1})^{\mathsf {T}}& (\mathbf{h}^{2}_{1})^{\mathsf {T}}& (\mathbf{h}^{3}_{1})^{\mathsf {T}}\end{bmatrix}^{\mathsf {T}}_{(4L+2L^{o}+4)\times 1},\\ \mathbf{C}_{2} = \begin{bmatrix} \mathbf{H}_{2}^{1}& \mathbf{0}_{(2L+1)\times (2L^{o}+1)}\\ \mathbf{H}_{2}^{2} & \mathbf{0}_{(2L+1)\times (2L^{o}+1)} \\ \mathbf{0}_{(2L^{o}+1)\times (2L+1)} & \mathbf{H}_{2}^{3} \end{bmatrix}_{(4L+2L^{o}+3) \times (2L+2L^{o}+2)}, \end{cases}\displaystyle \end{aligned}$$

26 $$\begin{aligned} &\textit{Sampling vectors} \\ &\quad \leftarrow \textstyle\begin{cases} \boldsymbol{\varPhi}^{nmk} = \begin{bmatrix} 1\\ \mathbf{u}^{nmk} \\ -q^{nmk}\mathbf{u}^{nmk} \\ -q^{nmk} \mathbf{v}^{nmk} \end{bmatrix}_{(4L+2L^{o}+4) \times 1}, \\ \boldsymbol{\varXi}^{nmk} = \begin{bmatrix} \mathbf{U}_{2}^{nmk} & \mathbf{0}_{(2L+1)\times (2L^{o}+1)} \\ -q^{nmk}\mathbf{U}^{nmk}_{2} & \mathbf{0}_{(2L+1)\times (2L^{o}+1)} \\ \mathbf{0}_{(2L^{o}+1)\times (2L+1)} & -q^{nmk} \mathbf{V}_{2}^{nmk} \end{bmatrix}_{(4L+2L^{o}+3) \times (2L+2L^{o}+2)}. \end{cases}\displaystyle \end{aligned}$$

#### Proof

See Appendix 3 for more notation and detailed proof. □

A necessary condition on the number of trials and the number of measurements required for identifying the divisive normalization processor for solving the system of equations in Theorem 1 is that the number of trials $M \ge 3 + 2 \cdot \dim ({\mathcal {H}}_{1})$ and the number of total samples $TM \ge 1 + 2 \cdot \dim ({\mathcal {H}}_{1}) + 2 \cdot \dim ({\mathcal {H}}_{1})^{2}$.

It is easy to see that solving the system of equations above suffers from the curse of dimensionality. As the dimension of ${\mathcal {H}}_{1}$ increases, the number of samples needed to identify components increases quadratically. Note that the second-order Volterra kernels $h_{2}^{l}$, $l = 1,2,3$, have unique symmetric forms with orthogonal expansions as follows [57]:

27 $$ h_{2}^{l}(t_{1},t_{2}) = \sum _{k\in {\mathbb {N}}} \lambda ^{kl} g^{kl}_{1}(t_{1})g^{kl}_{1}(t_{2}), \quad \bigl\Vert g_{1}^{kl} \bigr\Vert = 1 , $$

where $g_{1}^{kl} \in {\mathcal {H}}_{1}, k \in {\mathbb {N}}$, are orthogonal to each other. In what follows, we assume that the second-order Volterra kernels are sparse, *i.e.*, $\lambda ^{kl}=0$ for $k>r_{l}$, where $r_{l} \ll \dim ({\mathcal {H}}_{1})$. Sparse kernels often arise in modeling sensory processing, *e.g.*, in complex cells in the primary visual cortex [42]. By exploiting the sparse structure of the second-order kernels, the identification problem can be made tractable.

The sparsity of the kernels can be translated into a low-rank condition on the matrix representation of $h^{l}_{2}$, $l=1,2,3$ (see also Appendix 3). Ideally, the optimization problem would be a rank minimization problem. But rank minimization being NP-hard, we use the surrogate of nuclear norm minimization instead, which is the convex envelope of the rank operator [63].

To perform sparse identification of the divisive normalization processor, we devised Algorithm 1. By optimizing over $\mathbf{c}_{1}$ and $\mathbf{C}_{2}$ and subsequently assigning the corresponding block entries according to (25), Algorithm 1 identifies $b_{1}$, $\mathbf{h}^{i}_{1}$, $i= 1,2,3$, and $\mathbf{H}^{i}_{2}$, $i =1,2,3$.

As a surrogate of rank minimization, Algorithm 1 minimizes a linear combination of the nuclear norm of $\mathbf{C}_{2}$ and the Euclidean norm of $\mathbf{c}_{1}$. The optimization constraints correspond (i) in (29) to the generalized sampling problem allowing certain amount of error, (ii) in (30) to zero mean slack variables, (iii) in (31) to the zeros in the two blocks in the top-right of $\mathbf{C}_{2}$, (iv) in (32) to the zeros in the block in the lower-left of $\mathbf{C}_{2}$, and (v) in (33), (34), and (35), respectively, $\mathbf{H}^{1}_{2}$, $\mathbf{H}^{2}_{2}$, and $\mathbf{H}^{3}_{2}$ are Hermitian.

#### Algorithm 1

$\widehat{\mathbf{c}}_{1}$ and $\widehat{\mathbf{C}}_{2}$ are the solution to the following optimization problem:

28 $$\underset{c_{1},C_{2},\varepsilon }{\text{minimize}}{∥C_{2}∥}_{*}+\lambda_{1}{∥c_{1}∥}_{2}+\lambda_{2}{∥\varepsilon ∥}_{2}$$

29 $$\begin{array}{rlrl} & \text{s.t.} & & c_{1}^{T}\Phi^{nmk}+\mathrm{Tr}\left(C_{2}^{H}\Xi^{nmk}\right)=q^{nmk}+\varepsilon^{(m-1)*T+k},\\ & & & \;m=1,\ldots ,M,k=1,\ldots ,T, \end{array}$$

30 $$1^{T}\varepsilon =0$$

31 $$\left[\begin{array}{cc} I_{2\dim(H_{1})} & 0 \end{array}\right]C_{2}\left[\begin{array}{c} 0\\ I_{\dim(H_{1}^{o})} \end{array}\right]=0,$$

32 $$\left[\begin{array}{cc} 0 & I_{\dim(H_{1}^{0})} \end{array}\right]C_{2}\left[\begin{array}{c} I_{\dim(H_{1})}\\ 0 \end{array}\right]=0,$$

33 $$\left[\begin{array}{cc} I_{\dim(H_{1})} & 0 \end{array}\right]C_{2}\left[\begin{array}{c} I_{\dim(H_{1})}\\ 0 \end{array}\right]={\left[\begin{array}{c} I_{\dim(H_{1})}\\ 0 \end{array}\right]}^{T}{C_{2}}^{H}{\left[\begin{array}{cc} I_{\dim(H_{1})} & 0 \end{array}\right]}^{T},$$

34 $$\begin{array}{rlrl} & & & \left[\begin{array}{ccc} 0_{\dim(H_{1})} & I_{\dim(H_{1})} & 0 \end{array}\right]C_{2}\left[\begin{array}{c} I_{\dim(H_{1})}\\ 0 \end{array}\right]\\ & & & \;={\left[\begin{array}{c} I_{\dim(H_{1})}\\ 0 \end{array}\right]}^{T}{C_{2}}^{H}{\left[\begin{array}{ccc} 0_{\dim(H_{1})} & I_{\dim(H_{1})} & 0 \end{array}\right]}^{T}, \end{array}$$

35 $$\left[\begin{array}{cc} 0 & I_{\dim(H_{1}^{o})} \end{array}\right]C_{2}\left[\begin{array}{c} 0\\ I_{\dim(H_{1}^{o})} \end{array}\right]={\left[\begin{array}{c} 0\\ I_{\dim(H_{1}^{o})} \end{array}\right]}^{T}{C_{2}}^{H}{\left[\begin{array}{cc} 0 & I_{\dim(H_{1}^{o})} \end{array}\right]}^{T},$$

where $\|\cdot \|_{*}$ denotes the nuclear norm defined as $\|\mathbf{C}_{2}\|_{*} = \operatorname{Tr} ( (\mathbf{C}_{2}^{\mathsf {H}}\mathbf{C}_{2} )^{\frac{1}{2}} )$, $\lambda _{1}, \lambda _{2}$ are appropriately chosen hyperparameters, $\varepsilon^{i} \in \mathbb{R}$, $i = 1,2,\ldots,MT$, represent slack variables, **1** represents a vector of all ones, $\mathbf{I}_{p}$ represents a $p\times p$ identity matrix, $\mathbf{0}_{p}$ represents a $p\times p$ matrix of all zeros, $\mathbf{0}_{p\times q}$ represents a $p\times q$ matrix of all zeros, and **0** represents a matrix of all zeros with dimensions that make the equation consistent.

#### Theorem 1

*The filters of the DNP are identified as* $\hat{b}^{1} = \hat{b}^{1}$ *and*

36 $$\begin{aligned} &\widehat{h_{1}^{1}} (t) = \sum _{l = -L}^{L} \bigl[\widehat{\mathbf{h}^{1}_{1}} \bigr]_{l+L+1} \cdot e_{l}(t), \end{aligned}$$

37 $$\begin{aligned} & \widehat{h_{2}^{1}} (t_{1}, t_{2}) = \sum _{l_{1} = -L}^{L}\sum _{l_{2} = -L}^{L} \bigl[\widehat{\mathbf{H}^{1}_{2}} \bigr]_{l_{1}+L+1,L+1-l_{2}} \cdot e_{l_{1}}(t_{1}) e_{l_{2}}(t_{2}), \end{aligned}$$

38 $$\begin{aligned} &\widehat{h_{1}^{2}} (t) = \sum _{l = -L}^{L} \bigl[\widehat{\mathbf{h}^{2}_{1}} \bigr]_{l+L+1} \cdot e_{l}(t), \end{aligned}$$

39 $$\begin{aligned} & \widehat{h_{2}^{2}} (t_{1}, t_{2}) = \sum _{l_{1} = -L}^{L}\sum _{l_{2} = -L}^{L} \bigl[\widehat{\mathbf{H}^{2}_{2}} \bigr]_{l_{1}+L+1,L+1-l_{2}} \cdot e_{l_{1}}(t_{1}) e_{l_{2}}(t_{2}), \end{aligned}$$

40 $$\begin{aligned} &\widehat{h_{1}^{3}} (t) = \sum _{l = -L^{o}}^{L^{o}} \bigl[\widehat{\mathbf{h}^{3}_{1}} \bigr]_{l+L+1} \cdot e^{o}_{l}(t), \end{aligned}$$

41 $$\begin{aligned} & \widehat{h_{2}^{3}} (t_{1}, t_{2}) = \sum _{l_{1} = -L^{o}}^{L^{o}} \sum _{l_{2} = -L^{o}}^{L^{o}} \bigl[\widehat{\mathbf{H}^{3}_{2}} \bigr]_{l_{1}+L^{o}+1,L^{o}+1-l_{2}} \cdot e^{o}_{l_{1}}(t_{1}) e^{o}_{l_{2}}(t_{2}), \end{aligned}$$

*where*

42 $$\begin{aligned} \begin{bmatrix} \hat{b}^{1} & \widehat{\mathbf{h}^{1}_{1}}^{\mathsf {T}}& \widehat{\mathbf{h}^{2}_{1}}^{\mathsf {T}}& \widehat{\mathbf{h}^{3}_{1}}^{\mathsf {T}}\end{bmatrix}^{\mathsf {T}}= \widehat{\mathbf{c}}_{1} \quad \textit{and}\quad \begin{bmatrix} \widehat{\mathbf{H}_{2}^{1}}& - \\ \widehat{\mathbf{H}_{2}^{2}} & - \\ - & \widehat{\mathbf{H}_{2}^{3}} \end{bmatrix} = \widehat{ \mathbf{C}}_{2} . \end{aligned}$$

#### Remark 4

By exploiting the structure of low-rank second-order Volterra kernels, Algorithm 1 provides a tractable solution to the identification of the components of the divisive normalization processor.

### Examples of sparse identification of temporal DNPs

We provide here identification examples solved using Algorithm 1.

#### Example 1

Here, we identify a temporal divisive normalization processor in Fig. 2, where

$$\begin{aligned}& h^{1}_{1}(t) = 2.472\times 10^{10} t^{3} e^{-100\pi t}\cos (36\pi t), \\& h^{2}_{1}(t) = 3.117\times 10^{8} t^{3} e^{-100\pi t} \cos (20\pi t), \\& h^{3}_{1}(t) = 4.753\times 10^{8} t^{3} e^{-100\pi t} \cos (52 \pi t), \\& h^{1}_{2}(t,s) = 9.038 \times 10^{19} t^{3}s^{3} e^{-100\pi (t+s)} \cos (52\pi t)\cos (52\pi s) \\& \hphantom{h^{1}_{2}(t,s) =}{}+ 5.3467\times 10^{14} t^{3}s^{3} e^{-100\pi (t+s)} \cos (100\pi t)\cos (100\pi s), \\& h^{2}_{2}(t,s) = 1.533 \times 10^{19} t^{3}s^{3} e^{-100\pi (t+s)} \cos (68\pi t)\cos (68\pi s) \\& \hphantom{h^{2}_{2}(t,s) =}{}+ 5.970\times 10^{14} t^{3}s^{3} e^{-100\pi (t+s)} \cos (84\pi t)\cos (84\pi s), \\& h^{3}_{2}(t,s) = 6.771 \times 10^{19} t^{3}s^{3} e^{-100\pi (t+s)} \cos (100\pi t)\cos (100\pi s) \\& \hphantom{h^{3}_{2}(t,s) =}{}+ 5.970\times 10^{16} t^{3}s^{3} e^{-100\pi (t+s)} \cos (84\pi t)\cos (84\pi s). \end{aligned}$$

**Figure 4:**
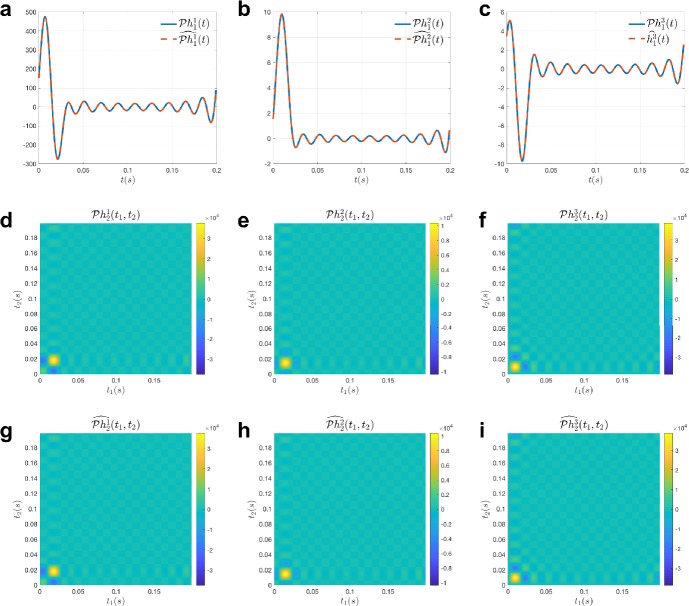
Example of identification of a divisive normalization model. (**a**) $\mathcal{P}h^{1}_{1}$ (blue) and $\widehat{\mathcal{P}h^{1}_{1}}$ (red, SNR 60.56 [dB]), (**b**) $\mathcal{P}h^{2}_{1}$ (blue) and $\widehat{\mathcal{P}h^{2}_{1}}$ (red, SNR 60.48 [dB]), (**c**) $\mathcal{P}h^{3}_{1}$ (blue) and $\widehat{\mathcal{P}h^{3}_{1}}$ (red, SNR 49.56 [dB]), (**d**) $\mathcal{P}h^{1}_{2}$, (**e**) $\mathcal{P}h^{2}_{2}$, (**f**) $\mathcal{P}h^{3}_{2}$, (**g**) $\widehat{\mathcal{P}h^{1}_{2}}$ (SNR 60.59 [dB]), (**h**) $\widehat{\mathcal{P}h^{2}_{2}}$ (SNR 60.54 [dB]), (**i**) $\widehat{\mathcal{P}h^{3}_{2}}$ (SNR 60.61 [dB])

We choose the input space ${\mathcal {H}}_{1}$ to have $L = 10, \varOmega = 100\pi $. Thus $S = 0.2s$, $\dim ({\mathcal {H}}_{1}) = 21$, and $\dim ({\mathcal {H}}_{2}) = 441$. Note that all three second-order Volterra kernels exhibit low-rank structure. We presented the model with 25 stimuli from ${\mathcal {H}}_{1}$, whose coefficients were chosen to be i.i.d Gaussian variables. Then, a total of 425 measurements were used from the input and the observed output pairs to solve the identification problem using Algorithm 1. The results of the identification are shown in Fig. 4. As can be seen from the figure, Algorithm 1 was able to identify the model with high precision using only 425 measurements, much less than the 1387 measurements that would have been required to solve the generalized sampling problem directly. The factor of reduction in the required measurements is critical when the model needs to be identified in a much larger space, for example, a space of spatio-temporal stimuli as shown in the next example.

#### Example 2

Here, we identify a detailed biophysical model of Drosophila photoreceptors as a DNP with feedforward only (see Fig. 2). The detailed biophysical model consists of 30,000 microvilli [5, 64]. The photon absorption in each microvillus is described by a Poisson process whose rate is proportional to the number of photons per microvillus incident on the ommatidium. Photon absorption leads to a transduction process governed by a cascade of chemical reactions. The entire transduction process is described by 13 successive differential equations and is given in [64]. The total number of equations of the photoreceptor model is $390,000$.

**Figure 5:**
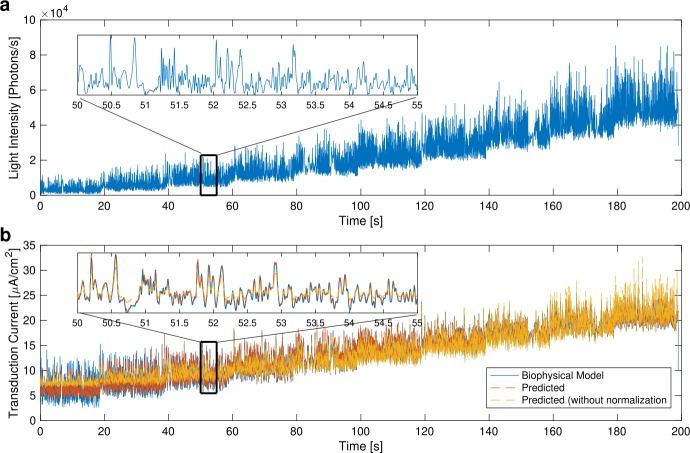
Output of the identified DNP model of the photoreceptor. (**a**) The stimulus presented to the photoreceptor; 5% of the stimulus was used for identification. (**b**) Comparison of the output of the detailed biophysical photoreceptor model (blue) with the output of the DNP model (red) and the output of the model without normalization, i.e., with $\mathcal{T}_{1}$ only (yellow)

For identifying the DNP, we used natural images from the van Hateren database [65] and simulated movements of a fly superimposed on the natural scenes. Each visual scene was captured by a previously developed retina model [64], endowed with realistic optics and the geometry of the fly retina. A 200-second stimulus was generated. We then divided the stimulus into several segments, each of which scaled to a different level of mean light intensity. The resulting stimulus is shown in Fig. 5(a). The bandwidth of the DNP input and output spaces was limited to 50 Hz.

**Figure 6:**
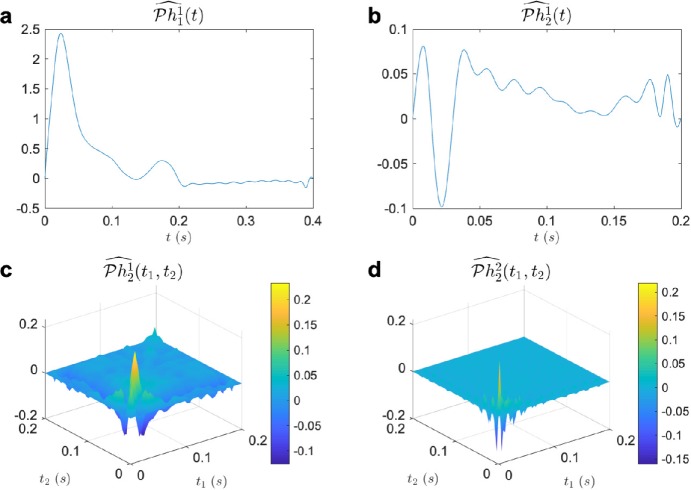
Identified filters of the DNP model of the photoreceptor given in Example 2. (**a**) $\widehat{\mathcal{P}h^{1}_{1}}$, (**b**) $\widehat{\mathcal{P}h^{2}_{1}}$, (**c**) $\widehat{\mathcal{P}h^{1}_{2}}$, (**d**) $\widehat{\mathcal{P}h^{2}_{2}}$

The visual input was presented to the DNP photoreceptor model. We used 10 seconds of the stimulus for identifying the DNP filters. The identified filters are depicted in Fig. 6. The output of the photoreceptor DNP model when stimulated by the other 190 seconds of the input stimulus is shown in Fig. 5(b). We evaluated the identified photoreceptor DNP model by computing the SNR between the output of the detailed biophysical model and that of the identified DNP model. The SNR was 26.14 [dB].

We additionally trained a model without normalization (*i.e.*, with only $\mathcal{T}^{1}$). As shown in Fig. 5(b) the identified model without normalization does not match the output well across different light intensities. Compared with the output of the DNP model, the SNR was only 14.48 [dB].

**Figure 7:**
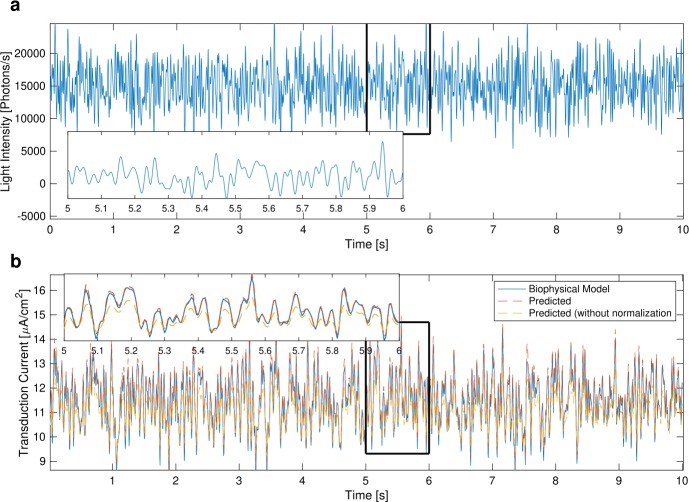
Evaluation of the DNP model of the photoreceptor. (**a**) Gaussian noise stimulus with a bandwidth of 50 Hz. (**b**) Output of the photoreceptor model (blue) and of the identified DNP model (red, SNR 15.04 [dB]). The output of the identified model without normalization is shown in yellow (SNR 4.55 [dB])

The DNP filters were identified using naturalistic stimuli. To test if the identified filters can also match the output of other types of stimuli, we presented a Gaussian noise stimulus with a bandwidth of 50 Hz to the photoreceptor model. The resulting output was compared with the output of the detailed biophysical model. As shown in Fig. 7, the output of the DNP model closely follows the actual photoreceptor output, and the SNR was 15.04 [dB]. We note that since the input space is defined as an RKHS, the statistics of the input stimuli do not affect the quality of the DNP output. We also evaluated in Fig. 7 the identification model without normalization. The SNR of the output is 4.55 [dB], a significant decrease from the output of the DNP model.

## Sparse identification of spatio-temporal DNPs

In what follows we derive sparse identification algorithms for the components of spatio-temporal DNPs.

Given the spatio-temporal divisive normalization processor depicted in Fig. 3, we are interested in identifying all the filters from input and output observations. We formulate an optimization problem, which achieves such identification, with high fidelity and with a relatively small number of measurements.

### Deriving the sparse identification algorithm for spatio-temporal DNPs

Here, we make the assumption that the filters $h^{i4}(t), i = 1,2,\ldots ,N$, and $h_{2}^{ij4}(t_{1}, t_{2})$, $i,j = 1,2,\ldots ,N$, are followed by the LPF with bandwidth $\varOmega ^{o}$ and that the output is sampled at a rate of $2f_{\max }$, where $f_{\max } = \frac{\varOmega }{2\pi }$ is the maximum of the bandwidth of the filters $h^{i4}(t)$ and $h_{2}^{ij4}(t_{1}, t_{2})$. By abuse of notation, we will use $v^{i}(t)$ to denote the low-passed version of the actual output. Note that, based upon the assumptions on the bandlimited nature of the feedback filters acting on the output, the responses of these filters to the low-passed outputs will be the same as the responses to the actual outputs.

We present *M* trials where input stimuli $u^{nm}$ are chosen to be elements of the space of trigonometric polynomials ${\mathcal {H}}_{1}$ for $n=1, \ldots , N$, $m=1, \ldots ,M$. We project the outputs $v^{nm}(t)$ on the Hilbert space of trigonometric polynomials ${\mathcal {H}}_{1}^{o}$, i.e., $\mathcal{P}_{1}^{o} v^{nm} (s)$. Note that $\mathcal{P}_{1}^{o} v^{nm} (s)$ is approximately $v^{nm} (s)$ for large values of $\varOmega ^{o}$ [66]. Further we assume that $v^{nm}, h^{1}_{1}, h^{2}_{1}, h^{3}_{1}, h^{i4}_{1}\in \mathbb{L}^{2}(\mathbb{D})$, $i = 1,2,\ldots ,N$, the space of square integrable functions over domain $\mathbb{D}$ and $h_{2}^{1}, h_{2}^{2}, h_{2}^{3}, h_{2}^{ij4} \in \mathbb{L}^{2}(\mathbb{D}^{2})$, $i,j = 1,2,\ldots ,N$, the space of square integrable functions over domain $\mathbb{D}^{2}$.

We consider here the identification of the entire DNP circuit at once for two reasons. First, as all channels are connected in the spatial domain through the MVP, the inputs to the MVP are the outputs of the entire DNP circuit. Therefore, all outputs are required to identify the MVP. Second, since $h^{i}_{1}, h^{i}_{2}$, $i=1,2,3$, are shared across all channels, fewer trials are needed to identify these filters. We present the following.

#### Lemma 3

*WithMtrials presented to the spatio*-*temporal DNP*, *let the inputs*$u^{nm}(t)$*and the outputs*$v^{nm}(t)$*be sampled at times*$(t_{k})$, $k = 1, \ldots , T$. *Then*, *for*$i,j=1,2,\ldots ,N$, *we have the following equations*:

43 $$\begin{aligned} &b^{1}+ \bigl\langle h_{1}^{1}, \phi _{11}^{nmk} \bigr\rangle _{{\mathcal {H}}_{1}} + \bigl\langle h_{1}^{2} , \phi _{12}^{nmk} \bigr\rangle _{{\mathcal {H}}_{1}} + \bigl\langle h_{1}^{3} , \phi _{13}^{nmk} \bigr\rangle _{{\mathcal {H}}_{1}^{o}} + \sum _{i=1}^{N} \bigl\langle h_{1}^{i4} , \phi _{14}^{inmk} \bigr\rangle _{{\mathcal {H}}_{1}^{o}} \\ &\qquad {}+ \bigl\langle h_{2}^{1}, \phi _{21}^{nmk} \bigr\rangle _{{\mathcal {H}}_{2}} + \bigl\langle h_{2}^{2}, \phi _{22}^{nmk} \bigr\rangle _{{\mathcal {H}}_{2}} + \bigl\langle h_{2}^{3}, \phi _{23}^{nmk} \bigr\rangle _{{\mathcal {H}}_{2}^{o}} + \sum _{i=1}^{N} \sum _{j=1}^{N} \bigl\langle h_{2}^{ij4} , \phi _{24}^{ijnmk} \bigr\rangle _{{\mathcal {H}}_{2}^{o}} \\ &\quad = q^{nmk} , \end{aligned}$$

*where the sampling functions*$\phi _{11}^{nmk} \in {\mathcal {H}}_{1}$, $\phi _{12}^{nmk}\in \mathcal{H}_{1}$, $\phi _{13}^{nmk}\in \mathcal{H}_{1}^{o}$, $\phi _{14}^{inmk}\in \mathcal{H}_{1}^{o}$, $\phi _{21}^{nmk}\in \mathcal{H}_{2}$, $\phi _{22}^{nmk}\in \mathcal{H}_{2}$, $\phi _{23}^{nmk}\in \mathcal{H}_{2}^{o}$, *and*$\phi _{24}^{ijnmk}\in \mathcal{H}_{2}^{o}$*are given by*

44 $$\begin{aligned}& \begin{gathered} \phi _{11}^{nmk} (t) = u^{nm}(t_{k}-t) , \\ \phi _{12}^{nmk} (t) = -q^{nm}(t_{k}) u^{nm}(t_{k}-t) , \\ \phi _{13}^{nmk} (t) = -q^{nm}(t_{k}) \bigl(\mathcal{P}_{1}^{o} v^{nm}\bigr) (t_{k}-t) , \\ \phi _{14}^{inmk} (t) = -q^{nm}(t_{k}) \bigl(\mathcal{P}_{1}^{o} v^{im}\bigr) (t_{k}-t) , \\ \phi _{21}^{nmk} (t, s) = u^{nm}(t_{k}-t)u^{nm}(t_{k}-s) , \\ \phi _{22}^{nmk} (t, s) = -q^{nm}(t_{k}) u^{nm}(t_{k}-t)u^{nm}(t_{k}-s) , \\ \phi _{23}^{nmk} (t, s) = -q^{nm}(t_{k}) \bigl(\mathcal{P}_{1}^{o} v^{nm}\bigr) (t_{k}-t) \bigl(\mathcal{P}_{1}^{o} v^{nm}\bigr) (t_{k}-s) , \\ \phi _{24}^{ijnmk} (t, s) = -q^{nm}(t_{k}) \bigl(\mathcal{P}_{1}^{o} v^{im}\bigr) (t_{k}-t) \bigl(\mathcal{P}_{1}^{o} v^{jm}\bigr) (t_{k}-s) , \end{gathered} \end{aligned}$$

*and*$q_{k}^{nm} = v^{nm}(t_{k})$*for all*$m=1,2,\ldots,M$, $k=1,2,\ldots,T$, *and*$i,j,n=1,2,\ldots,N$.

#### Proof

See Appendix 4. □

#### Remark 5

Theorem 3 suggests that identifying the lateral divisive normalization model is equivalent to solving a generalized sampling problem with noisy measurements. It also suggests that the output needs to be sampled at a high enough rate, and that the choice of the Hilbert space used to reconstruct the feedback filters is critical since incorrect choices for these parameters can negatively affect the identification by introducing ‘noise’ in the measurements.

We now present the following algorithm to identify the model that exploits the low-rank constraints imposed on the quadratic filters.

#### Lemma 4

*Equation* (43) *can be expressed in matrix form as follows*:

45 $$ \mathbf{c}_{1}^{\mathsf {T}}\boldsymbol{\varPhi}^{nmk} + \operatorname{Tr} \bigl(\mathbf{C}_{2}^{\mathsf {H}}\boldsymbol {\varXi}^{nmk} \bigr) = q^{nmk}, $$

*for all*$n=1,2,\ldots ,N$, $m=1,2,\ldots ,M$, *and*$k=1,2,\ldots ,T$, *where*

46 $$\begin{aligned} & \textit{Measurements} \leftarrow q^{mk} (scalar) \end{aligned}$$

47 Unknowns←{c1=[b1(h11)T(h12)T(h13)Tc1=[(h114)T(h124)T⋯(h1N4)T]1×(4L+2(N+1)Lo+N+4))T,C2=[(H21)T(H22)T0000(H23)T(H2114)TC2=[0⋯0(H2124)T⋯(H2NN4)T](2L+2Lo+2)×(4L+2(N2+1)Lo+N2+3))T,

48 $$\begin{aligned} & \textit{Sampling matrices} \\ &\quad \leftarrow \textstyle\begin{cases} \boldsymbol{\varPhi}^{nmk} = \begin{bmatrix} 1 \\ \mathbf{u}^{nmk} \\ -q^{nmk}\mathbf{u}^{nmk} \\ -q^{nmk} \mathbf{v}^{nmk} \\ -q^{nmk} \mathbf{v}^{1mk} \\ -q^{nmk} \mathbf{v}^{2mk} \\ \vdots \\ -q^{nmk} \mathbf{v}^{Nmk} \end{bmatrix}_{ (4L+2(N+1)L^{o}+N+4) ) \times 1}, \\ \boldsymbol{\varXi}^{nmk} = \begin{bmatrix} \mathbf{U}_{2}^{nmk} & \mathbf{0} \\ -q^{nmk}\mathbf{U}_{2}^{nmk} & \mathbf{0} \\ \mathbf{0} & -q^{nmk} \mathbf{V}_{2}^{nnmk} \\ \mathbf{0} & -q^{nmk} \mathbf{V}_{2}^{11mk} \\ \mathbf{0} & -q^{nmk} \mathbf{V}_{2}^{12mk} \\ \vdots & \vdots \\ \mathbf{0} & -q^{nmk} \mathbf{V}_{2}^{NNmk} \end{bmatrix}_{ (4L+2(N^{2}+1)L^{o}+N^{2}+3) ) \times (2L+2L^{o}+2 )} . \end{cases}\displaystyle \end{aligned}$$

We provide the following algorithm to identify $b_{1}$, $\mathbf{h}^{i}_{1}$, $i= 1,2,3$, $\mathbf{h}^{i4}_{1}$, $i= 1,2,\ldots ,N$, $\mathbf{H}^{i}_{2}$, $i =1,2,3$, and $\mathbf{H}^{ij4}_{2}$, $i,j =1,2,\ldots ,N$.

Again, we assume that all the second-order filters have sparse structures akin to (27).

#### Algorithm 2

Let $\widehat{\mathbf{c}}_{1}$ and $\widehat{\mathbf{C}}_{2}$ be the solution to the following optimization problem:

49 $$\min_{c_{1},C_{2},\varepsilon }{∥C_{2}∥}_{*}+\lambda_{1}{∥c_{1}∥}_{2}+\lambda_{2}{∥\varepsilon ∥}_{2}$$

50 $$\begin{array}{rlrl} & \;s.t & & c_{1}^{T}\Phi^{nmk}+\mathrm{Tr}\left(C_{2}^{H}\Xi^{nmk}\right)=q^{nmk}+\varepsilon^{(n-1)TM+(m-1)T+k},\\ & & & \;n=1,\ldots ,N,m=1,\ldots ,M,k=1,\ldots ,T, \end{array}$$

51 $$1^{T}\varepsilon =0$$

52 $$\left[\begin{array}{cc} I_{2\dim(H_{1})} & 0 \end{array}\right]C_{2}\left[\begin{array}{c} 0\\ I_{\dim(H_{1}^{o})} \end{array}\right]=0,$$

53 $$\left[\begin{array}{cc} 0 & I_{\dim(H_{1}^{0})} \end{array}\right]C_{2}\left[\begin{array}{c} I_{\dim(H_{1})}\\ 0 \end{array}\right]=0,$$

54 $$\left[\begin{array}{cc} I_{\dim(H_{1})} & 0 \end{array}\right]C_{2}\left[\begin{array}{c} I_{\dim(H_{1})}\\ 0 \end{array}\right]={\left[\begin{array}{c} I_{\dim(H_{1})}\\ 0 \end{array}\right]}^{T}{C_{2}}^{H}{\left[\begin{array}{cc} I_{\dim(H_{1})} & 0 \end{array}\right]}^{T},$$

55 $$\begin{array}{rlrl} & & & \left[\begin{array}{ccc} 0_{\dim(H_{1})} & I_{\dim(H_{1})} & 0 \end{array}\right]C_{2}\left[\begin{array}{c} I_{\dim(H_{1})}\\ 0 \end{array}\right]\\ & & & \;={\left[\begin{array}{c} I_{\dim(H_{1})}\\ 0 \end{array}\right]}^{T}{C_{2}}^{H}{\left[\begin{array}{ccc} 0_{\dim(H_{1})} & I_{\dim(H_{1})} & 0 \end{array}\right]}^{T}, \end{array}$$

56 $$\left[\begin{array}{cc} 0 & I_{\dim(H_{1}^{o})} \end{array}\right]C_{2}\left[\begin{array}{c} 0\\ I_{\dim(H_{1}^{o})} \end{array}\right]={\left[\begin{array}{c} 0\\ I_{\dim(H_{1}^{o})} \end{array}\right]}^{T}{C_{2}}^{H}{\left[\begin{array}{cc} 0 & I_{\dim(H_{1}^{o})} \end{array}\right]}^{T},$$

where $\lambda _{1}, \lambda _{2}$ are appropriately chosen hyperparameters and $\varepsilon^{i} \in \mathbb{R}$, $i=1,2,\ldots,MTN$, represent slack variables.

The constraints in Algorithm 2 are similar to those in Algorithm 1. Note that $H^{ij4}$ are not constrained to be Hermitian. This follows from the assumption that the MVP block may perform asymmetric processing on any pair of inputs to the block.

#### Theorem 2

*The identified spatio*-*temporal divisive normalization is specified as*$\hat{b}^{1} = \hat{b}^{1}$*and*

57 $$\begin{aligned} &\widehat{h_{1}^{1}} (t) = \sum _{l = -L}^{L} \bigl[\widehat{\mathbf{h}^{1}_{1}} \bigr]_{l+L+1} \cdot e_{l}(t), \qquad \widehat{h_{1}^{2}} (t) = \sum _{l = -L}^{L} \bigl[\widehat{ \mathbf{h}^{2}_{1}}\bigr]_{l+L+1} \cdot e_{l}(t), \end{aligned}$$

58 $$\begin{aligned} &\widehat{h_{1}^{3}} (t) = \sum _{l = -L^{o}}^{L^{o}} \bigl[\widehat{\mathbf{h}^{3}_{1}} \bigr]_{l+L+1} \cdot e^{o}_{l}(t), \qquad \widehat{h_{1}^{i4}} (t) = \sum _{l_{=} -L^{o}}^{L^{o}} \bigl[\widehat{\mathbf{h}^{i4}_{1}} \bigr]_{l+L+1} \cdot e^{o}_{l}(t), \end{aligned}$$

59 $$\begin{aligned} & \widehat{h_{2}^{1}} (t_{1}, t_{2}) = \sum _{l_{1} = -L}^{L}\sum _{l_{2} = -L}^{L} \bigl[\widehat{\mathbf{H}^{1}_{2}} \bigr]_{l_{1}+L+1,L+1-l_{2}} \cdot e_{l_{1}}(t_{1}) e_{l_{2}}(t_{2}), \end{aligned}$$

60 $$\begin{aligned} & \widehat{h_{2}^{2}} (t_{1}, t_{2}) = \sum _{l_{1} = -L}^{L}\sum _{l_{2} = -L}^{L} \bigl[\widehat{\mathbf{H}^{2}_{2}} \bigr]_{l_{1}+L+1,L+1-l_{2}} \cdot e_{l_{1}}(t_{1}) e_{l_{2}}(t_{2}), \end{aligned}$$

61 $$\begin{aligned} & \widehat{h_{2}^{3}} (t_{1}, t_{2}) = \sum _{l_{1} = -L^{o}}^{L^{o}}\sum _{l_{2} = -L^{o}}^{L^{o}} \bigl[\widehat{\mathbf{H}^{3}_{2}} \bigr]_{l_{1}+L^{o}+1,L^{o}+1-l_{2}} \cdot e^{o}_{l_{1}}(t_{1}) e^{o}_{l_{2}}(t_{2}), \end{aligned}$$

62 $$\begin{aligned} & \widehat{h_{2}^{ij4}} (t_{1}, t_{2}) = \sum _{l_{1} = -L^{o}}^{L^{o}}\sum _{l_{2} = -L^{o}}^{L^{o}} \bigl[\widehat{\mathbf{H}^{ij4}_{2}} \bigr]_{l_{1}+L^{o}+1,L^{o}+1-l_{2}} \cdot e^{o}_{l_{1}}(t_{1}) e^{o}_{l_{2}}(t_{2}), \\ & \quad i,j=1,2,\ldots ,N , \end{aligned}$$

*where*

63 $$\begin{aligned} \begin{bmatrix} \hat{b}^{1} & \widehat{\mathbf{h}^{1}_{1}}^{\mathsf {T}}& \widehat{\mathbf{h}^{2}_{1}}^{\mathsf {T}}& \widehat{\mathbf{h}^{3}_{1}}^{\mathsf {T}}& \widehat{\mathbf{h}^{14}_{1}}^{\mathsf {T}}& \widehat{\mathbf{h}^{24}_{1}}^{\mathsf {T}}& \cdots & \widehat{\mathbf{h}^{N4}_{1} }^{\mathsf {T}}\end{bmatrix}^{\mathsf {T}}= \widehat{\mathbf{c}}_{1} \end{aligned}$$

*and*

64 $$\begin{aligned} \begin{bmatrix} \widehat{\mathbf{H}_{2}^{1}}^{\mathsf {T}}& \widehat{\mathbf{H}_{2}^{2}}^{\mathsf {T}}& - & - & - & \cdots & - \\ - & - & \widehat{\mathbf{H}_{2}^{3}}^{\mathsf {T}}& \widehat{\mathbf{H}_{2}^{114}}^{\mathsf {T}}& \widehat{\mathbf{H}_{2}^{124}}^{\mathsf {T}}& \cdots & \widehat{\mathbf{H}_{2}^{NN4}}^{\mathsf {T}}\end{bmatrix}^{\mathsf {T}}= \widehat{\mathbf{C}}_{2} . \end{aligned}$$

#### Remark 6

Assuming that all the second-order kernels in the lateral divisive normalization model have rank *r*, the expected number of measurements for Algorithm 2 to identify the model is of the order $\mathcal{O} (r \cdot \dim ({\mathcal {H}}_{1}) + N^{2}r \cdot \dim ({\mathcal {H}}_{1}^{o}) )$. When *N* is large, the $N^{2}$ factor may become prohibitive in identifying the model. Additional assumptions on $h^{ij4}_{2}$ may help mitigate this and maintain tractability of solving the identification problem. For example, with the assumption that $h^{ij4} = h^{14}_{1}$ if $i=j$ and $h^{ij4}_{2} = h^{24}_{1}$ otherwise, the expected number of measurements required will be $\mathcal{O} (r \cdot \dim ({\mathcal {H}}_{1}) + Nr \cdot \dim ({\mathcal {H}}_{1}^{o}) )$.

### An example of sparse identification of a spatio-temporal DNP

We now present an example of identification obtained by Algorithm 2. We demonstrate here that, in addition to the identification of the Volterra kernels operating within each channel, the MVP in the spatio-temporal DNP can be identified.

#### Example 3

Here, we choose the DNP in Fig. 3 with $N=4$, and

65 $$\begin{aligned}& h^{1}_{1}(t) = 25 t e^{-25t}, \end{aligned}$$

66 $$\begin{aligned}& h^{2}_{1}(t) = 25te^{-25t}, \end{aligned}$$

67 $$\begin{aligned}& h^{i4}_{1}(t) = e^{ -\frac{1}{4}(i-2)^{2}}(25-600t)e^{-25t}, \end{aligned}$$

68 $$\begin{aligned}& h^{ij4}_{2}(t_{1},t_{2}) = 5000 e^{ -\frac{1}{4}(i-2)^{2}}e^{ -\frac{1}{4}(j-2)^{2}} \cdot \bigl(25t_{1}e^{-25t_{1}} \bigr) \bigl(25t_{2}e^{-25t_{2}}\bigr), \end{aligned}$$

and the other Volterra kernels are set to 0. Note that Yiyin, and $h^{1}_{1}$, $h^{2}_{1}$ are shared across all channels. In addition, $h^{ij4} = h^{ji4}$ in this case. We assumed knowledge of this symmetric structure of the model and adapted the identification algorithm accordingly and only identified six linear filters and ten quadratic filters.

**Figure 8:**
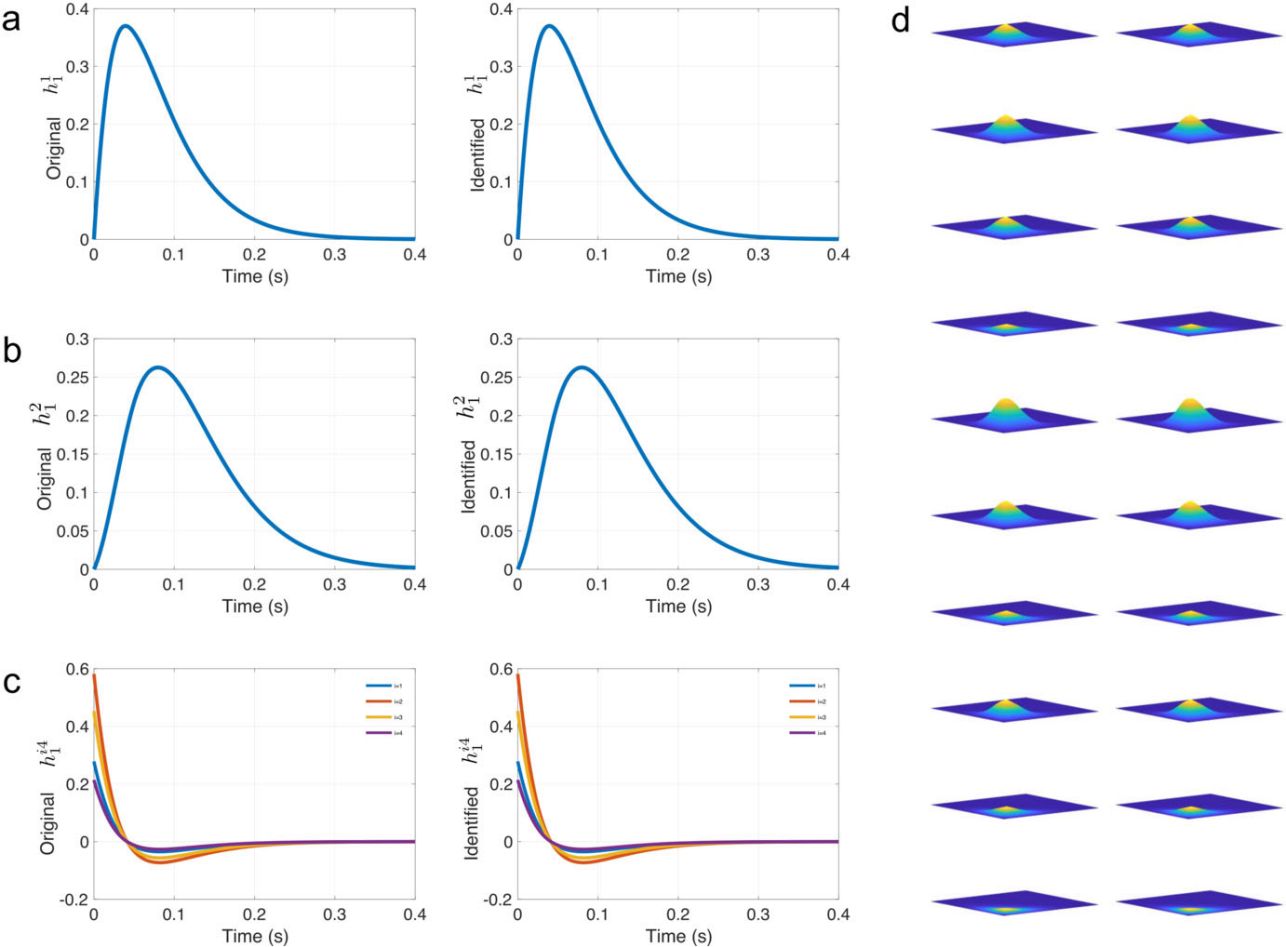
Example of identification of the spatio-temporal DNP given in Example 3. (**a**)–(**c**) Identification of the first-order filters (**a**) $h_{1}^{1}$, (**b**) $h_{1}^{2}$, and (**c**) $h_{1}^{i4}$, $i=1,2,3,4$. (**d**) Identification of the second-order filters (from top to bottom) $h_{2}^{ij4}$ with $i \leq j$ for $i=1,2,3,4$ and $j=1,2,3,4$

**Figure 9:**
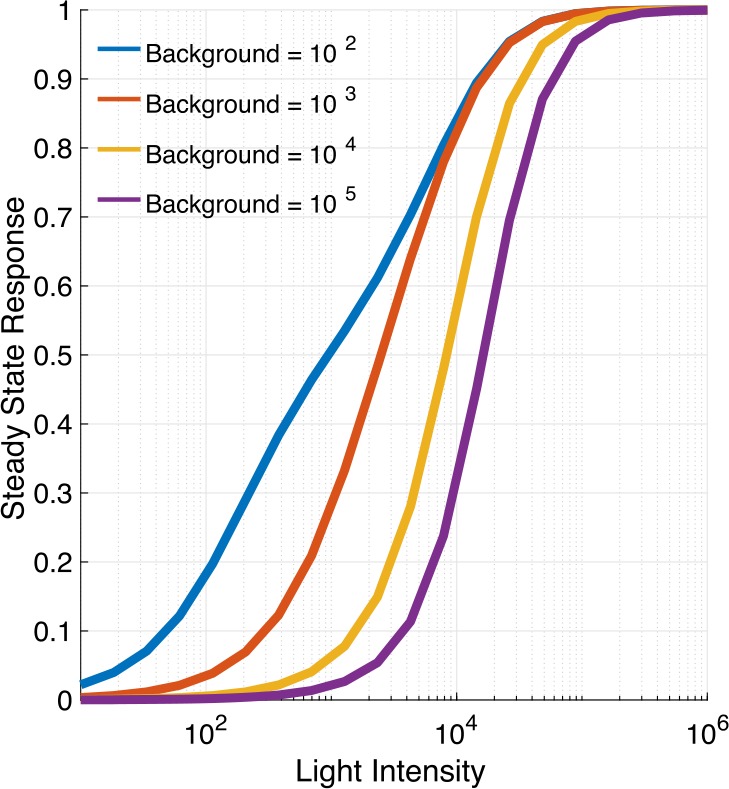
The spatio-temporal DNP model can exhibit adaptation to local luminance. A DNP with four photoreceptors and an amacrine cell (see Example 1 for details) is adapted to a background intensity level at the beginning of each trial. One of the photoreceptors is then provided a two-second long flash of light, while the inputs to the other photoreceptors are kept at the background level. The relationship between the steady state response of the photoreceptor and the light intensity of the flash is shown for each of the background levels

**Figure 10:**
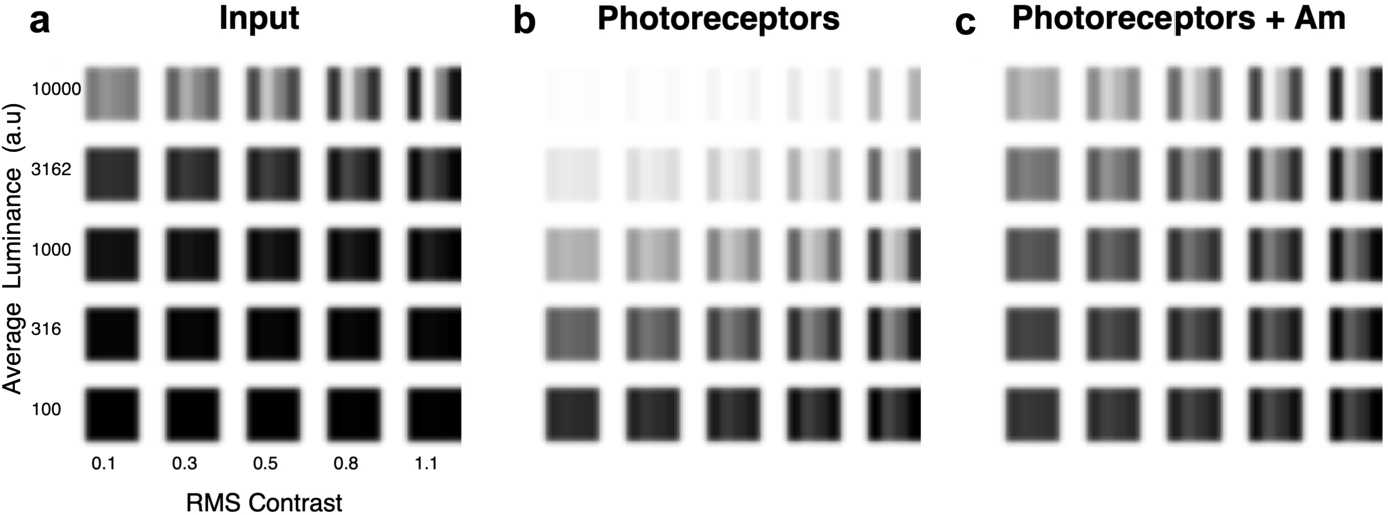
Steady State I/O visualization for the DNP considered in Example 1. (**a**) Input stimuli comprising bar gratings at various luminances and RMS contrasts. (**b**) Responses of the DNP without the MVP block. (**c**) Responses of the DNP with the MVP block

**Figure 11:**
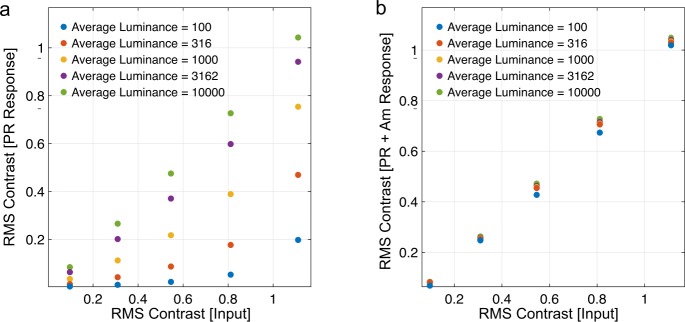
RMS contrast of the input images in Figure 10 are plotted against the RMS contrasts of the responses (**a**) without and (**b**) with the MVP block

**Figure 13:**
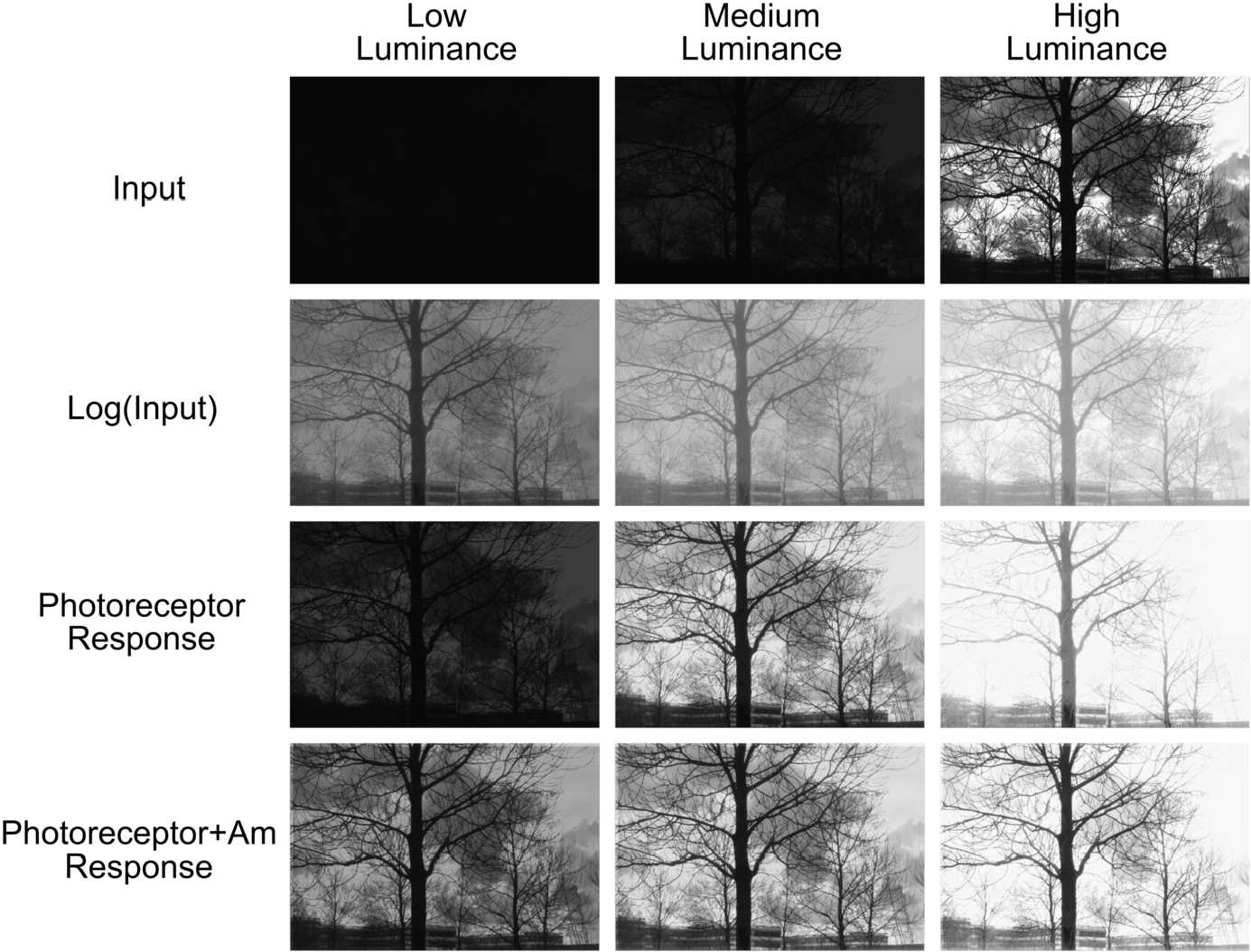
Steady state I/O visualization of the spatio-temporal DNP for natural images. Top Row—Input images. Second Row—Input images visualized on a logarithmic scale. Third Row—Responses without the MVP block. Fourth Row—Responses with the MVP block. Left Column—Stimuli presented at low luminance, Middle Column—Medium luminance, Right Column—High luminance

We performed the identification in the Hilbert space of bandlimited functions with $\varOmega = \varOmega ^{o} = 40\pi $, and we chose the same space for the input stimuli and for both the feedforward filters and the feedback ones. We solved for the filters truncated to a period of 0.4 s, and thus there were 17 coefficients to be identified for the linear filters and $17\times 17$ coefficients to be identified for each of the quadratic filters. A total of 1116 measurements (279 measurements from each cell) were used to perform the identification, and the results are depicted in Fig. 8. The average SNR of reconstruction across all filters was more than 150 [dB]. Note that solving the generalized sampling problem directly for the same problem would have required at least 3570 measurements.

## Discussion

As already mentioned in the Introduction, the photoreceptor/amacrine cell layer of the early vision system of the fruit fly rapidly adapts to visual stimuli whose intensity and contrast vary orders of magnitude both in space and time.

In this paper we presented a spatio-temporal divisive normalization processor that models the transduction and the contrast gain control in the photoreceptor and amacrine cell layer of the fruit fly. It incorporates processing blocks that explicitly model the feedforward and the temporal feedback path of each photoreceptor and the spatio-temporal feedback from amacrine cells to photoreceptors. We demonstrated that with some simple choice of parameters, the DNP response maintains the contrast of the input visual field across a large range of average spatial luminance values.

We characterized the I/O of the spatio-temporal DNP and highlighted the highly nonlinear behavior of the DNP in contrast gain control. Despite the divisive nonlinearity, we provided an algorithm for the sparse identification of the entire DNP. We showed that the identification of the DNP components can be interpreted as a generalized sampling problem. More importantly, the sparse identification algorithm does not suffer from the curse of dimensionality that would otherwise require a large number of measurements that are quadratically related to the dimension of the input and output spaces.

Neural circuits are often noisy. Although the I/O characterization of MIMO DNPs provided in Sect. 2 is noise free, it can be easily extended to account for noise [52–54]. The identification of DNPs can be interpreted as a generalized sampling problem in an RKHS with noisy measurements. The sparse identification algorithms we provided in this paper include slack variables to account for these inaccurate measurements. Similar algorithms have been shown to be robust to noise [42].

Contrast gain control is an intrinsically nonlinear problem. Early approaches to modeling contrast gain control rely on analyzing the Volterra kernels, *i.e.*, linear or nonlinear receptive fields identified when stimuli of different statistics are presented [19, 20, 23]. The objective of these studies is to observe and characterize the differences between the identified Volterra kernels. To fully capture the nonlinear effect in contrast gain control, these approaches would rely on identifying higher-order Volterra kernels that are too costly to compute and are simply ignored. The contrast gain control model advanced in this paper, however, results from the divisive normalization operation, albeit having Volterra kernels modeling internal filters in the DNP. The division largely expands upon DNP’s ability to model higher-order nonlinearities in contrast gain control.

Biologically, divisive normalization can take place in feedforward or feedback circuits, or both [26]. Most divisive normalization models only consider the feedforward normalization circuit. Although normalization can be achieved by a multiplicative feedback mechanism, it mainly serves as a transformation leading to an equivalent form of feedforward divisive normalization when the input is a constant [27, 67]. The MIMO DNP models described in this paper explicitly include divisive normalization with both feedforward and feedback paths that can be more readily employed in modeling underlying neuronal circuit mechanisms, such as those arising in the photoreceptor and amacrine cell layer.

Predictive coding often takes the form subtractive negative feedback [68]. The MIMO DNP models described in this paper represent an alternative form of predictive coding. Indeed, divisive input modulation has been proposed to implement predictive coding by calculating the residual errors using division rather than subtraction [69]. In flies, it has been suggested that inhibition in the lamina, where retina photoreceptors and amacrine cells interact, leads to predictive coding [70]. Our study advances a spatio-temporal MIMO model for predictive coding and provides an algorithm to identify the components of the MIMO DNP.

The MIMO DNP model opens a new avenue for exploring and quantifying the highly nonlinear nature of sensory processing. The MIMO DNP in Fig. 3 can be further extended to allow the different transformations $\mathcal{T}^{i}$, $i=1,2,3$, to incorporate spatio-temporal Volterra kernels, thereby making it more versatile for modeling other types of sensory processing, including (i) interactions between cones and horizontal cells in vertebrate retinas [58], (ii) channels/glomeruli in olfactory circuits and interactions between them through local neurons [9, 71], and (iii) cross-suppression and gain control in the auditory [12, 72], and visual cortices [73–75]. The sparse identification algorithms advanced here can be easily extended to identify the MIMO DNPs of these systems as well.

## Figures and Tables

**Figure 12:**
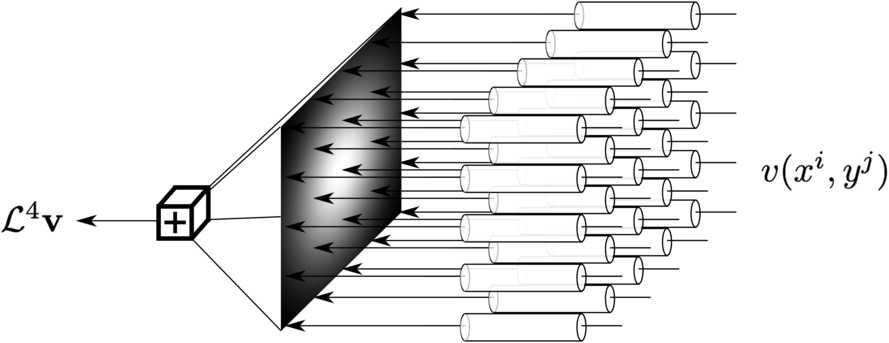
Graphical visualization of the feedback processor of the DNP used in Example 6. The value of the feedback is obtained by a weighted sum of the outputs at each pixel of a $16\times 16$ block (only five pixels are shown in each direction) processed by a temporal Volterra operator (represented by a cylinder). Each weight is the value of a Gaussian function evaluated at the distance between the location of the pixel and the center of the block

## Appendix 1: Spatio-temporal DNPs and contrast gain control

Here, we characterize the I/O of simple DNPs stimulated with three different inputs. In the first example, we evaluate the response of a $1 \times 4$ DNP under different background light intensity levels. In the second example, contrast gain control exerted by the amacrine cells is demonstrated with the same DNP. In the third example, a DNP consisting of $16 \times 16$ DNPs tiling a $1536 \times 1024$ visual field is stimulated with a natural image taken at low, medium, and high luminance values. The DNP output is evaluated with and without the MVP block.

### Example 4

Here, we consider a simple $1 \times 4$ DNP consisting of four photoreceptors and a single amacrine cell receiving inputs from and providing feedback to all four photoreceptors. The choices of the component filters of the DNP are as follows:

69 $$ b_{2}+b_{3}+b_{4} = 1, $$

for the transformations $\mathcal{T}^{1}$ and $\mathcal{T}^{2}$, the kernels are

70 $$\begin{aligned}& h_{1}^{1}(t) = h_{1}^{2}(t) = \frac{t}{0.1} \operatorname{exp} \biggl(-\frac{t}{0.1} \biggr), \end{aligned}$$

71 $$\begin{aligned}& h_{2}^{1}(t_{1},t_{2}) = h_{2}^{2}(t_{1},t_{2}) = 0.0001 h_{1}^{1}(t_{1})h_{1}^{1}(t_{2}), \end{aligned}$$

for the transformation $\mathcal{T}^{3}$, the kernels are

72 $$\begin{aligned}& h_{1}^{3}(t) = 0, \end{aligned}$$

73 $$\begin{aligned}& h_{2}^{3}(t_{1},t_{2}) = 0, \end{aligned}$$

and for the transformation $\mathcal{L}^{4}$, the kernels are

74 $$\begin{aligned}& h^{i4}_{1}(t) = 10\mbox{,}000 \frac{t}{0.8} \operatorname{exp} \biggl(-\frac{t}{0.2} \biggr),\quad i = 1,2,3,4, \end{aligned}$$

75 $$\begin{aligned}& h^{ij4}_{2}(t_{1},t_{2}) = \textstyle\begin{cases} 12\mbox{,}500\frac{t_{1} t_{2}}{0.04} \operatorname{exp} (-\frac{t_{1}+t_{2}}{0.2} ), & i = j,\\ 0, & i \neq j, \end{cases}\displaystyle \quad i, j = 1,2,3,4. \end{aligned}$$

Here, the bandwidth of ${\mathcal {H}}^{o}_{1}$ was chosen to be $\varOmega ^{o} = 10\cdot 2\pi $ rad/s. The I/O of the fly’s individual photoreceptors in steady state is described by a saturating nonlinearity similar in appearance to a sigmoid when plotted on a logarithmic scale [1]. Photoreceptors can deal with the orders of magnitude of the input stimuli while maintaining their output in a suitable range. In addition, the photoreceptors exhibit adaptation so that the sigmoidal curves shift to adjust to the mean local luminance value [26].

We examine here the steady state response of the above DNP under four different background light intensity levels. At the start of each trial, all four photoreceptors are first adapted to the same background light intensity level. One of the photoreceptors is then subjected to an additional flash of different light intensity with a duration of two seconds, while the inputs to the other three are kept at the same background level. We observe the value of the steady state response of the photoreceptor that receives the additional flash. Figure 9 depicts the relationship between the observed steady state response and the light intensity of the flash at the four background intensity levels. It demonstrates that the response of the DNP is background-dependent, and the overall slope and range are similar across different background levels. With the MVP block, the spatio-temporal DNP can reproduce responses of photoreceptors observed in experimental settings [26, 46].

Since all the photoreceptors exhibit a sigmoid like nonlinearity, the output of the retina will be constrained in a suitable current range that can be processed by postsynaptic neurons in the lamina. However, without adaptation to mean local luminance, the saturating nature of the nonlinearities leads to a loss of spatial contrast. The spatial contrast is preserved by spatial gain control or adaptation modeled here with the MVP block.

### Example 5

Here, the I/O of DNPs with and without the MVP block is evaluated. Using the same DNP as in the example above, we stimulated the DNP with 25 “images” with a resolution of $1\times 4$ pixels. Each image has a different average luminance and root mean square (RMS) contrast. The RMS contrast is defined as the standard deviation of pixel intensities normalized by the mean, i.e.,

76 $$ C_{rms} = \dfrac{\sqrt{\frac{1}{N} \sum_{i=1}^{N} (u^{i} - \overline{u} )^{2}}}{\overline{u}}, $$

where

77 $$ \overline{u} = \frac{1}{N} \sum_{i=1}^{N} u^{i} $$

and $N=4$. These images are shown in the “input” block in Fig. 10, with each bar representing the input intensity to one photoreceptor. Note that the pixels are extended along the y-axis for a quick visual examination.

In the “Photoreceptors” block in Fig. 10, the steady state responses of the DNP without the MVP block to the respective inputs are shown. Here, pure black and white represent a response of 0 and 1, respectively. This can be interpreted as a circuit in which the reciprocal connections between photoreceptors and amacrine cells are blocked.

In the “Photoreceptors + Am” block in Fig. 10, the steady state responses of the full DNP to their respective inputs are shown. Comparing the “Photoreceptors” and “Photoreceptors + Am” blocks, the responses of the DNP without feedback are washed out, or exhibit low contrast, particularly at three of the four corners of the $5\times 5$ image array, *i.e.*, when either the luminance or contrast is too high or too low. By contrast, the individual bars in the response of the full DNP model are more readily discernible across several orders of magnitude of luminance.

We note that a saturating nonlinearity can maintain its output in a constrained range even when the input varies by orders of magnitude. However, the nonlinearity may lead to a loss of spatial contrast. In contrast, the DNP constrains its output to a suitable range while maintaining the spatial contrast. This is demonstrated in Fig. 11, where the RMS contrast of the input images in Fig. 10 is plotted against the RMS contrasts of the responses for both cases—with and without MVP in the DNP. Spatial contrast, arguably, is an important feature of the image that should be preserved or even enhanced for subsequent stages of extraction of ethologically relevant information from the visual stimulus.

### Example 6

Here, we apply a full-scale DNP model to a natural image. The image is taken in raw format so that its pixel values are proportional to the light intensity each pixel is exposed to [65]. The resolution of the image is $1536\times 1024$. The image is first divided into $16\times 16$ blocks with a four-pixel overlap in each direction. A DNP is assigned to each block, and the filters in the DNP are designed as follows:

78 $$ b_{2}+b_{3}+b_{4} = 1, $$

for the transformations $\mathcal{T}^{1}$ and $\mathcal{T}^{2}$, the kernels are

79 $$\begin{aligned}& h_{1}^{1}(t) = h_{1}^{2}(t) = \frac{t}{0.04} \operatorname{exp} \biggl(-\frac{t}{0.04} \biggr), \end{aligned}$$

80 $$\begin{aligned}& h_{2}^{1}(t_{1},t_{2}) = h_{2}^{2}(t_{1},t_{2}) = 0.0001 h_{1}^{1}(t_{1})h_{1}^{1}(t_{2}), \end{aligned}$$

for the transformation $\mathcal{T}^{3}$, the kernels are

81 $$\begin{aligned}& h_{1}^{3}(t) = 0, \end{aligned}$$

82 $$\begin{aligned}& h_{2}^{3}(t_{1},t_{2}) = 0, \end{aligned}$$

and for the transformation $\mathcal{L}^{4}$, the kernels are

83 $$\begin{aligned}& h^{i4}_{1}(t) = 20 \frac{t}{0.04} \operatorname{exp} \biggl(-\frac{t}{0.04} \biggr) \cdot \frac{1}{\sqrt{2\pi \cdot 16}}\operatorname{exp} \biggl( - \frac{(x^{i}-x^{0})^{2}+(y^{i}-y^{0})^{2}}{32} \biggr) , \\ & \quad i = 1,2,\ldots ,256, \end{aligned}$$

84 $$\begin{aligned}& \begin{aligned} &h^{ij4}_{2}(t_{1},t_{2}) = \textstyle\begin{cases} 100\frac{t_{1} t_{2}}{0.04^{2}} \operatorname{exp} (-\frac{t_{1}+t_{2}}{0.04} ) \cdot \sqrt{\frac{1}{\sqrt{2\pi \cdot 16}}\operatorname{exp} ( - \frac{(x^{i}-x^{0})^{2}+(y^{i}-y^{0})^{2}}{32} )} , & i = j,\\ 0, & i \neq j, \end{cases}\displaystyle \\ &\quad i, j = 1,2,\ldots ,256, \end{aligned} \end{aligned}$$

where $(x^{i}, y^{i})$ is the coordinate of pixel *i*, and $(x^{0}, y^{0})$ is the coordinate of the center pixel in the $16\times 16$ block. Here, the bandwidth of ${\mathcal {H}}^{o}_{1}$ was chosen to be $\varOmega ^{o} = 40\cdot 2\pi $ rad/s.

Figure 12 provides a graphical visualization of the feedback processing of the DNP. We note that the RHS of both equalities (83) and (84) can be separated into a temporal term and a spatial term. The temporal terms correspond to temporal Volterra kernels processing the output at each pixel (see cylinders in Fig. 12). The feedback term is then the weighted sum with a weight according to the spatial term. Each weight is the value of a Gaussian function evaluated at the distance between the location of the pixel and the center of the block. Due to the overlaps between blocks, there are pixels that belong to multiple blocks. The feedback term for such a pixel is the average of the feedback terms from all the blocks the pixel belongs to.

We tested the DNP using low, medium, and high luminance (1×, 10×, and 100×) of the original image measured in the number of photons and represented by arbitrary units. They are shown on the top row of Fig. 13. The logarithms of the inputs are shown on the second row. On the third row, we show the response of the DNP without the MVP block, *i.e.*, each photoreceptor processes independently a single pixel. The responses of the full DNP model are shown on the bottom row.

As can be seen from Fig. 13, the response of the DNP with MVP is robust across the different levels of luminance, showing the best quality for all three luminance levels. By contrast, the response of the DNP without MVP is either too dark for low contrast or saturated at high luminance. The contrast within each response is also significantly worse. It is also not ideal to use only a logarithmic nonlinearity to process the images (second row), as the contrast of the images is not sharp enough.

To conclude we note that, with a simple choice of filters, the spatio-temporal DNP proposed in Sect. 2.3 operates in a range of luminance values spanning several orders of magnitude, much like the photoreceptor-amacrine cell layer. The contrast of the output of the DNP is not compromised while its range remains strongly bounded.

## Appendix 2: Proof of Lemma 1

For the *m*th trial and the *k*th time sample, multiplying out the denominator in (5), we get

85 $$\begin{aligned} &v^{nm}(t_{k}) \biggl( 1 + \int _{\mathbb{D}}~h_{1}^{2}(s) u^{nm} (t_{k}-s) \,ds + \int _{\mathbb{D}^{2}}~h_{2}^{2}(s_{1},s_{2}) u_{2}^{nm}(t_{k}-s_{1}, t_{k}-s_{2}) \,ds_{1} \,ds_{2} \biggr) \\ &\qquad {}+ v^{nm}(t_{k}) \biggl( \int _{\mathbb{D}}~h_{1}^{3}(s) \bigl( \mathcal{P}_{1}^{o} v^{nm}\bigr) (t_{k}-s) \,ds \\ &\qquad {}+ \int _{\mathbb{D}^{2}}~h_{2}^{3} (s_{1},s_{2}) \bigl(\mathcal{P}_{2}^{o} v_{2}^{nm}\bigr) (t_{k}-s_{1}, t_{k}-s_{2}) \,ds_{1} \,ds_{2} \biggr) \\ &\quad = b_{1} + \int _{\mathbb{D}}~h_{1}^{1}(s) u^{nm}(t_{k}-s) \,ds + \int _{\mathbb{D}^{2}}~h_{2}^{1}(s_{1},s_{2}) u_{2}^{nm}(t_{k}-s_{1}, t_{k}-s_{2}) \,ds_{1} \,ds_{2} , \end{aligned}$$

where $u_{2}^{nm} (t_{1}, t_{2}) \in \mathcal{H}_{2}$ and $u_{2}^{nm} (t_{1}, t_{2}) = u^{nm} (t_{1})u^{nm} (t_{2})$.

With the notation in (22) and after rearranging terms in equation (85) above, we obtain

86 $$\begin{aligned} v^{nm}(t_{k}) ={}& b^{1}+ \int _{\mathbb{D}}~h_{1}^{1}(s) \phi _{11}^{nmk} \,ds + \int _{\mathbb{D}}~h_{1}^{2}(s) \phi _{12}^{nmk} \,ds + \int _{\mathbb{D}} ~h_{1}^{3}(s) \phi _{13}^{nmk} \,ds \\ &{}+ \int _{\mathbb{D}^{2}}~h_{2}^{1}(s_{1},s_{2}) \phi _{21}^{nmk} \,ds_{1} \,ds_{2} + \int _{\mathbb{D}^{2}}~h_{2}^{2}(s_{1},s_{2}) \phi _{22}^{nmk} \,ds_{1} \,ds_{2} \\ &{}+ \int _{\mathbb{D}^{2}}~h_{2}^{3}(s_{1},s_{2}) \phi _{23}^{nmk} \,ds_{1} \,ds_{2} . \end{aligned}$$

With $q^{nmk} = v^{nm}(t_{k})$, equation (86) becomes

$$\begin{aligned}& b^{1} + \bigl\langle h_{1}^{1}, \phi _{11}^{nmk} \bigr\rangle _{{\mathcal {H}}_{1}} + \bigl\langle h_{1}^{2} , \phi _{12}^{nmk} \bigr\rangle _{{\mathcal {H}}_{1}} + \bigl\langle h_{1}^{3} , \phi _{13}^{nmk} \bigr\rangle _{{\mathcal {H}}_{1}^{o}} \\& \quad {}+ \bigl\langle h_{2}^{1}, \phi _{21}^{nmk} \bigr\rangle _{{\mathcal {H}}_{2}} + \bigl\langle h_{2}^{2}, \phi _{22}^{nmk} \bigr\rangle _{{\mathcal {H}}_{2}} + \bigl\langle h_{2}^{3}, \phi _{23}^{nmk} \bigr\rangle _{{\mathcal {H}}_{2}^{o}} = q^{nmk} \end{aligned}$$

for all $m=1,2,\ldots,M$ and $k=1,2,\ldots,T$.

## Appendix 3: Proof of Lemma 2

Let

87 $$\begin{aligned}& h^{1}_{1} (t) = \sum_{l = -L}^{L} h^{1}_{1l} \cdot e_{l}(t), \qquad h^{1}_{2} (t) = \sum_{l_{1} = -L}^{L} \sum_{l_{2} = -L}^{L} h^{1}_{2 l_{1} l_{2}} \cdot e_{l_{1}}(t_{1}) \cdot e_{l_{2}}(t_{2}), \end{aligned}$$

88 $$\begin{aligned}& h^{2}_{1} (t) = \sum_{l = -L}^{L} h^{2}_{1l} \cdot e_{l}(t),\qquad h^{2}_{2} (t) = \sum_{l_{1} = -L}^{L} \sum_{l_{2} = -L}^{L} h^{2}_{2 l_{1} l_{2}} \cdot e_{l_{1}}(t_{1}) \cdot e_{l_{2}}(t_{2}), \end{aligned}$$

89 $$\begin{aligned}& h^{3}_{1} (t) = \sum_{l = -L^{o}}^{L^{o}} h^{3}_{1l} \cdot e^{o}_{l}(t), \qquad h^{3}_{2} (t) = \sum_{l_{1} = -L^{o}}^{L^{o}} \sum_{l_{2} = -L^{o}}^{L^{o}} h^{3}_{2 l_{1} l_{2}} \cdot e^{o}_{l_{1}}(t_{1}) \cdot e^{o}_{l_{2}}(t_{2}), \end{aligned}$$

and the $(2L+1)\times 1$ vectors

90 $$ \mathbf{h}^{1}_{1} = \begin{bmatrix} h^{1}_{1,-L} \\ h^{1}_{1,-L+1} \\ \vdots \\ h^{1}_{1,L} \end{bmatrix}, \qquad \mathbf{h}^{2}_{1} = \begin{bmatrix} h^{2}_{1,-L} \\ h^{2}_{1,-L+1} \\ \vdots \\ h^{2}_{1,L} \end{bmatrix}, $$

the $(2L^{o}+1)\times 1$ vector

91 $$ \mathbf{h}^{3}_{1} = \begin{bmatrix} h^{3}_{1,-L^{o}} \\ h^{3}_{1,-L^{o}+1} \\ \vdots \\ h^{3}_{1,L^{o}} \end{bmatrix}, $$

the $(2L+1)\times (2L+1)$ Hermitian matrices

92 $$\begin{aligned}& \mathbf{H}^{1}_{2} = \begin{bmatrix} h^{1}_{2,-L,L} & \cdots & h^{1}_{2,L,L} \\ \vdots & \ddots & \vdots \\ h^{1}_{2,-L,-L} & \cdots & h^{1}_{2,L,-L} \end{bmatrix}, \end{aligned}$$

93 $$\begin{aligned}& \mathbf{H}^{2}_{2} = \begin{bmatrix} h^{2}_{2,-L,L} & \cdots & h^{2}_{2,L,L} \\ \vdots & \ddots & \vdots \\ h^{2}_{2,-L,-L} & \cdots & h^{2}_{2,L,-L} \end{bmatrix}, \end{aligned}$$

and the $(2L^{o}+1)\times (2L^{o}+1)$ Hermitian matrix

94 $$ \mathbf{H}^{3}_{2} = \begin{bmatrix} h^{3}_{2,-L^{o},L^{o}} & \cdots & h^{3}_{2,L^{o},L^{o}} \\ \vdots & \ddots & \vdots \\ h^{3}_{2,-L^{o},-L^{o}} & \cdots & h^{3}_{2,L^{o},-L^{o}} \end{bmatrix}. $$

Further, let

95 $$\begin{aligned} \mathbf{u}^{nmk} &= \sqrt{S} \begin{bmatrix} a^{nm}_{-L} \cdot e_{-L}(t_{k})\\ a^{nm}_{-L+1} \cdot e_{-L+1}(t_{k})\\ \vdots \\ a^{nm}_{L} \cdot e_{L}(t_{k}) \end{bmatrix}, \qquad \mathbf{U}_{\mathbf{2}}^{nmk} = \mathbf{u}^{nmk} \bigl(\mathbf{u}^{nmk} \bigr)^{\mathsf {H}}, \end{aligned}$$

where $a^{nm}_{l}$ represents the coefficient of $u^{nm}(t)$ w.r.t. basis element $e_{l}$ in ${\mathcal {H}}_{1}$ such that $u^{nm} = \sum_{l=-L}^{L} a^{nm}_{l} e_{l}$, and $(\cdot )^{\mathsf {H}}$ represents conjugate transpose, and let

96 $$\begin{aligned} \mathbf{v}^{nmk} &= \sqrt{S^{o}} \begin{bmatrix} d^{nm}_{-L^{o}} \cdot e^{o}_{-L^{o}}(t_{k})\\ d^{nm}_{-L^{o}+1} \cdot e^{o}_{-L^{o}+1}(t_{k})\\ \vdots \\ d^{nm}_{L^{o}} \cdot e^{o}_{L^{o}}(t_{k}) \end{bmatrix}, \qquad \mathbf{V}_{\mathbf{2}}^{nmk} = \mathbf{v}^{nmk} \bigl(\mathbf{v}^{nmk} \bigr)^{\mathsf {H}}, \end{aligned}$$

where $d^{nm}_{l}$ represents the coefficient of $(\mathcal{P}_{1}^{o} v^{nm})$ w.r.t. basis element $e^{o}_{l}$ in ${\mathcal {H}}^{o}_{1}$ such that $(\mathcal{P}_{1}^{o} v^{nm}) = \sum_{l=-L^{o}}^{L^{o}} d^{nm}_{l} e^{o}_{l}$.

Finally, we define the $(4L+2L^{o}+4)\times 1$ vector

97 $$\begin{aligned} \boldsymbol{\varPhi}^{nmk} &= \begin{bmatrix} 1 \\ \mathbf{u}^{nmk} \\ -q^{nmk}\mathbf{u}^{nmk} \\ -q^{nmk} \mathbf{v}^{nmk} \end{bmatrix} \end{aligned}$$

and $(4L+2L^{o}+3)\times (2L+2L^{o}+2)$ matrix

98 $$\begin{aligned} \boldsymbol{\varXi}^{nmk} &= \begin{bmatrix} \mathbf{U}_{2}^{nmk} & \mathbf{0}_{(2L+1)\times (2L^{o}+1)} \\ -q^{nmk}\mathbf{U}^{nmk}_{2} & \mathbf{0}_{(2L+1)\times (2L^{o}+1)} \\ \mathbf{0}_{(2L^{o}+1)\times (2L+1)} & -q^{nmk} \mathbf{V}_{2}^{nmk} \end{bmatrix}. \end{aligned}$$

Finally, we define the $(4L+2L^{o}+4)\times 1$ vector

99 $$\begin{aligned} \mathbf{c}_{1} = \begin{bmatrix} b^{1} & (\mathbf{h}^{1}_{1})^{\mathsf {T}}& (\mathbf{h}^{2}_{1})^{\mathsf {T}}& (\mathbf{h}^{3}_{1})^{\mathsf {T}}\end{bmatrix}^{\mathsf {T}} \end{aligned}$$

and the $(4L+2L^{o}+3)\times (2L+2L^{o}+2)$ matrix

100 $$\begin{aligned} \mathbf{C}_{2} = \begin{bmatrix} \mathbf{H}_{2}^{1}& \mathbf{0}_{(2L+1)\times (2L^{o}+1)}\\ \mathbf{H}_{2}^{2} & \mathbf{0}_{(2L+1)\times (2L^{o}+1)} \\ \mathbf{0}_{(2L^{o}+1)\times (2L+1)} & \mathbf{H}_{2}^{3} \end{bmatrix} . \end{aligned}$$

The proof is based on two simple observations. First,

101 $$\begin{aligned} \bigl\langle h_{1}^{1}, \phi _{11}^{nmk} \bigr\rangle _{{\mathcal {H}}_{1}} =& \Biggl\langle \sum_{l=-L}^{L} h_{1l}^{1} \cdot e_{l} (t) , \sum _{l=-L}^{L} a_{l}^{nm} \cdot e_{l} (t_{k} - t) \Biggr\rangle _{{\mathcal {H}}_{1}} \\ =& \sum_{l=-L}^{L} h_{1l}^{1} a_{l}^{nm} \cdot \sqrt{S}e_{l} (t_{k}) = \bigl(\mathbf{h}^{1}_{1} \bigr)^{\mathsf {T}}\mathbf{u}^{nmk}, \end{aligned}$$

and therefore

$$ b^{1} + \bigl\langle h_{1}^{1}, \phi _{11}^{nmk} \bigr\rangle _{{\mathcal {H}}_{1}} + \bigl\langle h_{1}^{2} , \phi _{12}^{nmk} \bigr\rangle _{{\mathcal {H}}_{1}} + \bigl\langle h_{1}^{3} , \phi _{13}^{nmk} \bigr\rangle _{{\mathcal {H}}_{1}^{o}} = \mathbf{c}_{1}^{{\mathsf {T}}} \boldsymbol{\varPhi}^{nmk}. $$

Second,

102 $$\begin{aligned} \bigl\langle h_{2}^{1}, \phi _{21}^{nmk} \bigr\rangle _{{\mathcal {H}}_{2}} &= \Biggl\langle \sum _{l_{1}=-L}^{L} \sum_{l_{2}=-L}^{L} h_{2l_{1}l_{2}}^{1} \cdot e_{l_{1}} (t) \cdot e_{l_{2}} (s) , \sum_{l_{1}=-L}^{L} a_{l_{1}}^{nm} e_{l_{1}} (t_{k}-t) \sum_{l_{2}=-L}^{L} a_{l_{2}}^{nm} e_{l_{2}} (t_{k} -s) \Biggr\rangle _{{\mathcal {H}}_{2}} \\ &= \sum_{l_{1}=-L}^{L} \sum _{l_{2}=-L}^{L} h_{2l_{1}l_{2}}^{1} a_{l_{1}}^{nm} a_{l_{2}}^{nm} \cdot e_{l_{1}} (t_{k}) \cdot e_{l_{2}} (t_{k}) = \operatorname{Tr} \bigl(\bigl(\mathbf{H}_{2}^{1} \bigr)^{\mathsf {H}}\mathbf{U}_{2}^{nmk} \bigr) \end{aligned}$$

and, therefore,

103 $$ \bigl\langle h_{2}^{1}, \phi _{21}^{nmk} \bigr\rangle _{{\mathcal {H}}_{2}} + \bigl\langle h_{2}^{2}, \phi _{22}^{nmk} \bigr\rangle _{{\mathcal {H}}_{2}} + \bigl\langle h_{2}^{3}, \phi _{23}^{nmk} \bigr\rangle _{{\mathcal {H}}_{2}^{o}} = \operatorname{Tr} \bigl(\mathbf{C}_{2}^{\mathsf {H}} \boldsymbol{\varXi}^{nmk} \bigr) . $$

## Appendix 4: Proof of Lemma 3

For the *m*th trial, the output of the *n*th channel, by multiplying by the denominator of (20) with $v^{nm}$ sampled at times $t_{k}$, we obtain

104 $$\begin{aligned} &v^{nm}(t_{k}) \Biggl( 1+ \int _{\mathbb{D}} h_{1}^{2}(s)u^{nm}(t_{k}-s) \,ds + \int _{\mathbb{D}^{2}} h_{2}^{2}(s_{1}, s_{2})u^{nm}(t_{k}-s_{1}) u^{nm}(t_{k}-s_{2}) \,ds_{1} \,ds_{2} \\ &\qquad {}+ \int _{\mathbb{D}} h_{1}^{3}(s) \bigl( \mathcal{P}_{1}^{o} v^{nm}\bigr) (t_{k}-s) \,ds \\ &\qquad {} + \int _{\mathbb{D}^{2}} h_{2}^{3}(s_{1}, s_{2}) \bigl(\mathcal{P}_{1}^{o} v^{nm}\bigr) (t_{k}-s_{1}) \bigl( \mathcal{P}_{1}^{o} v^{nm}\bigr) (t_{k}-s_{2}) \,ds_{1} \,ds_{2} \\ &\qquad {}+ \sum_{i=1}^{N} \int _{\mathbb{D}} h_{1}^{i4}(s) \bigl( \mathcal{P}_{1}^{o} v^{im}\bigr) (t_{k}-s) \,ds \\ &\qquad {} + \sum_{i=1}^{N} \sum_{j=1}^{N} \int _{\mathbb{D}^{2}} h_{2}^{ij4}(s_{1}, s_{2}) \bigl(\mathcal{P}_{1}^{o} v^{im}\bigr) (t_{k}-s_{1}) \bigl( \mathcal{P}_{1}^{o} v^{jm}\bigr) (t_{k}-s_{2}) \,ds_{1} \,ds_{2} \Biggr) \\ &\quad = b^{1} + \int _{\mathbb{D}} h_{1}^{1}(s)u^{nm}(t_{k}-s) \,ds + \int _{\mathbb{D}^{2}} h_{2}^{1}(s_{1}, s_{2}) u^{nm}(t_{k}-s_{1}) u^{nm}(t_{k}-s_{2}) \,ds_{1} \,ds_{2} . \end{aligned}$$

Finally, with $v^{nm}(t_{k}) = q^{nmk}$ and the notation in (44), we get

105 $$\begin{aligned} q^{nmk} ={}& b^{1} + \int _{\mathbb{D}} h_{1}^{1}(s) \phi _{11}^{nmk} (s) \,ds + \int _{\mathbb{D}^{2}} h_{2}^{1}(s_{1}, s_{2}) \phi _{21}^{nmk} (s_{1}, s_{2}) \,ds_{1} \,ds_{2} \\ &{} + \int _{\mathbb{D}} h_{1}^{2}(s) \phi _{12}^{nmk} (s) \,ds + \int _{\mathbb{D}^{2}} h_{2}^{2}(s_{1}, s_{2}) \phi _{22}^{nmk} (s_{1},s_{2}) \,ds_{1} \,ds_{2} \\ &{} + \int _{\mathbb{D}} h_{1}^{3}(s) \phi _{13}^{nmk} (s) \,ds + \int _{\mathbb{D}^{2}} h_{2}^{3}(s_{1}, s_{2}) \phi _{23}^{nmk} (s_{1},s_{2}) \,ds_{1} \,ds_{2} \\ &{} + \sum_{i=1}^{N} \int _{\mathbb{D}} h_{1}^{i4}(s) \phi _{14}^{inmk} (s) \,ds + \sum_{i=1}^{N} \sum_{j=1}^{N} \int _{\mathbb{D}^{2}} h_{2}^{ij4}(s_{1}, s_{2}) \phi _{24}^{ijnmk} (s_{1},s_{2}) \,ds_{1} \,ds_{2} , \end{aligned}$$

and, therefore,

106 $$\begin{aligned} &b^{1}+ \bigl\langle h_{1}^{1}, \phi _{11}^{nmk} \bigr\rangle _{{\mathcal {H}}_{1}} + \bigl\langle h_{1}^{2} , \phi _{12}^{nmk} \bigr\rangle _{{\mathcal {H}}_{1}} + \bigl\langle h_{1}^{3} , \phi _{13}^{nmk} \bigr\rangle _{{\mathcal {H}}_{1}^{o}} + \sum _{i=1}^{N} \bigl\langle h_{1}^{i4} , \phi _{14}^{inmk} \bigr\rangle _{{\mathcal {H}}_{1}^{o}} \\ &\quad {}+ \bigl\langle h_{2}^{1}, \phi _{21}^{nmk} \bigr\rangle _{{\mathcal {H}}_{2}} + \bigl\langle h_{2}^{2}, \phi _{22}^{nmk} \bigr\rangle _{{\mathcal {H}}_{2}} + \bigl\langle h_{2}^{3}, \phi _{23}^{nmk} \bigr\rangle _{{\mathcal {H}}_{2}^{o}} + \sum _{i=1}^{N} \sum _{j=1}^{N} \bigl\langle h_{2}^{ij4} , \phi _{24}^{ijnmk} \bigr\rangle _{{\mathcal {H}}_{2}^{o}} = q^{nm}_{k} \end{aligned}$$

for all $m=1,2,\ldots,M$, $k+1,2,\ldots,T$, and $i,j,n=1,2,\ldots,N$.

## Appendix 5: Proof of Lemma 4

In addition to (87)–(94), let

107 $$\begin{aligned}& h^{i4}_{1} (t) = \sum_{l = -L^{o}}^{L^{o}} h^{i4}_{1l} e^{o}_{l}(t), \quad i = 1,2,\ldots ,N, \end{aligned}$$

108 $$\begin{aligned}& h^{ij4}_{2} (t) = \sum_{l_{1} = -L^{o}}^{L^{o}} \sum_{l_{2} = -L^{o}}^{L^{o}} h^{ij4}_{2 l_{1} l_{2}} e^{o}_{l_{1}}(t_{1})e^{o}_{l_{2}}(t_{2}), \quad i,j = 1,2,\ldots ,N, \end{aligned}$$

and the $(2L^{o}+1)\times 1$ vector

109 $$ \mathbf{h}^{i4}_{1} = \begin{bmatrix} h^{i4}_{1,-L^{o}} \\ h^{i4}_{1,-L^{o}+1} \\ \vdots \\ h^{i4}_{1,L^{o}} \end{bmatrix}, \quad i = 1,2,\ldots ,N, $$

and the $(2L^{o}+1)\times (2L^{o}+1)$ matrix

110 $$ \mathbf{H}^{ij4}_{2} = \begin{bmatrix} h^{ij4}_{2,-L^{o},L^{o}} & \cdots & h^{ij4}_{2,L^{o},L^{o}} \\ \vdots & \ddots & \vdots \\ h^{ij4}_{2,-L^{o},-L^{o}} & \cdots & h^{ij4}_{2,L^{o},-L^{o}} \end{bmatrix}, \quad i, j = 1,2,\ldots ,N. $$

Note that $\mathbf{H}^{ij4}_{2}, i,j = 1,2,\ldots ,N$, are not necessarily Hermitian matrices. Further, by abuse of notation, let the $(2L+1)\times 1$ vector

111 $$ \mathbf{u}^{nmk} = \sqrt{S} \begin{bmatrix} a^{nm}_{-L} \cdot e_{-L}(t_{k})\\ a^{nm}_{-L+1} \cdot e_{-L+1}(t_{k})\\ \vdots \\ a^{nm}_{L} \cdot e_{L}(t_{k}) \end{bmatrix}, \qquad \mathbf{U}_{\mathbf{2}}^{nmk} = \mathbf{u}^{nmk} \bigl(\mathbf{u}^{nmk} \bigr)^{\mathsf {H}}, $$

where $a^{nm}_{l}$ represents the coefficient of $u^{nm}(t)$ w.r.t. basis element $e_{l}$ in ${\mathcal {H}}_{1}$ such that $u^{nm}(t) = \sum_{l=-L}^{L} a^{nm}_{l} e_{l}$, and let the $(2L^{o}+1)\times 1$ vector

112 $$ \mathbf{v}^{nmk} = \sqrt{S^{o}} \begin{bmatrix} d^{nm}_{-L^{o}} \cdot e^{o}_{-L^{o}}(t_{k})\\ d^{nm}_{-L^{o}+1} \cdot e^{o}_{-L^{o}+1}(t_{k})\\ \vdots \\ d^{nm}_{L^{o}} \cdot e^{o}_{L^{o}}(t_{k}) \end{bmatrix}, \qquad \mathbf{V}_{\mathbf{2}}^{n_{1}n_{2}mk} = \mathbf{v}^{n_{2}mk} \bigl(\mathbf{v}^{n_{1}mk} \bigr)^{\mathsf {H}}, $$

where $d^{nm}_{l}$ represents the coefficient of $(\mathcal{P}_{1}^{o} v^{nm})$ w.r.t. basis element $e^{o}_{l}$ in ${\mathcal {H}}^{o}_{1}$ such that $(\mathcal{P}_{1}^{o} v^{nm}) = \sum_{l=-L^{o}}^{L^{o}} d^{nm}_{l} e^{o}_{l}$.

Additionally, we define the $(1+2(2L+1)+(N+1)(2L^{o}+1) )\times 1$ vector

113 $$\begin{aligned} & \boldsymbol{\varPhi}^{nmk} \\ &\quad = \bigl[ 1, \bigl(\mathbf{u}^{nmk}\bigr)^{\mathsf {T}} , -q^{nmk}\bigl(\mathbf{u}^{nmk}\bigr)^{\mathsf {T}} , -q^{nmk} \bigl(\mathbf{v}^{nmk}\bigr)^{\mathsf {T}} , -q^{nmk} \bigl(\mathbf{v}^{1mk}\bigr)^{\mathsf {T}} , \\ &\qquad {} -q^{nmk} \bigl(\mathbf{v}^{2mk} \bigr)^{\mathsf {T}} , \cdots , -q^{nmk} \bigl(\mathbf{v}^{Nmk} \bigr)^{\mathsf {T}}\bigr]^{\mathsf {T}} , \end{aligned}$$

and the $(2(2L+1)+(N^{2}+1)(2L^{o}+1) )\times (2L+1+2L^{o}+1 )$ matrix

114 $$ \boldsymbol{\varXi}^{nmk} = \begin{bmatrix} \mathbf{U}_{2}^{nmk} & \mathbf{0} \\ -q^{nmk}\mathbf{U}_{2}^{nmk} & \mathbf{0} \\ \mathbf{0} & -q^{nmk} \mathbf{V}_{2}^{nnmk} \\ \mathbf{0} & -q^{nmk} \mathbf{V}_{2}^{11mk} \\ \mathbf{0} & -q^{nmk} \mathbf{V}_{2}^{12mk} \\ \vdots & \vdots \\ \mathbf{0} & -q^{nmk} \mathbf{V}_{2}^{NNmk} \end{bmatrix}. $$

Finally, we define the $(1+2(2L+1)+(N+1)(2L^{o}+1) )\times 1$ vector

115 $$\begin{aligned} \mathbf{c}_{1} = \begin{bmatrix} b^{1} & (\mathbf{h}^{1}_{1})^{\mathsf {T}}& (\mathbf{h}^{2}_{1})^{\mathsf {T}}& (\mathbf{h}^{3}_{1})^{\mathsf {T}}& (\mathbf{h}^{14}_{1})^{\mathsf {T}}& (\mathbf{h}^{24}_{1})^{\mathsf {T}}& \cdots & (\mathbf{h}^{N4}_{1})^{\mathsf {T}}\end{bmatrix}^{\mathsf {T}}, \end{aligned}$$

and the $(2(2L+1)+(N^{2}+1)(2L^{o}+1) )\times (2L+1+2L^{o}+1 )$ matrix

116 $$\begin{aligned} \mathbf{C}_{2} = \begin{bmatrix} (\mathbf{H}_{2}^{1})^{\mathsf {T}}& (\mathbf{H}_{2}^{2})^{\mathsf {T}}& \mathbf{0} & \mathbf{0} & \mathbf{0} & \cdots & \mathbf{0} \\ \mathbf{0} & \mathbf{0} & (\mathbf{H}_{2}^{3})^{\mathsf {T}}& (\mathbf{H}_{2}^{114})^{\mathsf {T}}& (\mathbf{H}_{2}^{124})^{\mathsf {T}}& \cdots & (\mathbf{H}_{2}^{NN4})^{\mathsf {T}}\end{bmatrix}^{\mathsf {T}}. \end{aligned}$$

With the notation above, the proof follows the same steps as the proof of Lemma 2.

## Abbreviations

DNP: Divisive normalization processor
LMC: Large monopolar cells
LPF: Low-pass filter
MVP: Multi-input Volterra processor
RKHS: Reproducing kernel Hilbert space
RMS: Root mean square
SNR: Signal-to-noise ratio
